# The neurofilament derived-peptide NFL-TBS.40-63 enters in-vitro in human neural stem cells and increases their differentiation

**DOI:** 10.1371/journal.pone.0201578

**Published:** 2018-08-09

**Authors:** Kristell Barreau, Claudia Montero-Menei, Joël Eyer

**Affiliations:** 1 Laboratoire Micro et Nanomédecines Translationnelles, Inserm 1066, CNRS 6021, Institut de Recherche en Ingénierie de la Santé, Bâtiment IBS Institut de Biologie de la Santé, Université Angers, Centre Hospitalier Universitaire, Angers, France; 2 Centre de Recherche en Cancérologie et Immunologie, INSERM, Université de Nantes, Université Angers, Angers, France; Universidade de Sao Paulo Instituto de Quimica, BRAZIL

## Abstract

Regenerative medicine is a promising approach to treat neurodegenerative diseases by replacing degenerating cells like neurons or oligodendrocytes. Targeting human neural stem cells directly in the brain is a big challenge in such a strategy. The neurofilament derived NFL-TBS.40-63 peptide has recently been introduced as a novel tool to target neural stem cells. Previous studies showed that this peptide can be internalized by rat neural stem cells *in vitro* and *in vivo*, which coincided with lower proliferation and self-renewal capacity and increase of differentiation. In this study, we analyzed the uptake and potential effects of the NFL-TBS.40-63 peptide on human neural stem cells isolated from human fetuses. We showed that the peptide inhibits proliferation and the ability to produce neurospheres *in vitro*, which is consistent with an increase in cell adhesion and differentiation. These results confirm that the peptide could be a promising molecule to target and manipulate human neural stem cells and thus could serve as a strategic tool for regenerative medicine.

## Introduction

Although there has been a steady increase in incidence rates for neurodegenerative diseases, effective treatments are still lacking. Regenerative therapy could help to reduce symptoms and reverse disease progression by replacing degenerative cells. Neural stem cells (NSCs) have the capacity to self-renew, proliferate, migrate and differentiate into neurons, astrocytes or oligodendrocytes in the central nervous system, and have become key targets for regenerative therapy of neurodegenerative diseases [1]. Moreover, some studies showed that endogenous or exogenous NSCs are able to migrate to brain tumors to protect the microenvironment from the tumor [2]. Currently, preclinical studies investigate the therapeutic effect of fetal NSCs in neurodegenerative diseases like Amyotrophic Lateral Sclerosis (ALS). Indeed, fetal NSCs have been transplanted into the lumbar or cervical segments of the spinal cord of ALS patients during phase 1 clinical trials [3]. An increasing number of clinical studies use NSCs to treat neurology diseases like neurodegenerative diseases, spinal cord injury or brain tumors [4–7].

The potential source for cell therapy clinical studies in the central nervous system includes human NSCs from fetuses, adults or pluripotent stem cells [1, 4]. The main source of endogenous NSCs in the human adult brain is the subventricular and subgranular zones. These cells thus need to be isolated from fetal tissue or adults after biopsy, grown *in vitro* and then transplanted into patients [8]. This procedure is difficult due to the low availability of tissues from the brain and ethical challenges. Therefore, regenerative medicine could be improved by targeting NSCs directly in the brain (that will then migrate and differentiate into neurons or oligodendrocytes at the site of injury) without removing them from their endogenous environment.

A potential candidate for such an objective is the NFL-TBS.40-63 peptide, which corresponds to the sequence of the tubulin-binding site (TBS) located on the neurofilament light subunit (NFL) between amino acids 40 and 63 [9]. Previous works showed that this peptide targets glioblastoma cells specifically (when compared to healthy cells like neurons or astrocytes), leading to a reduction in their viability, proliferation, and migration. When injected in the intracerebral tumor, its volume is reduced by 70% after 24 days of treatment [10]. This peptide not only increases oligodendrocyte differentiation and maturation, but also protects oligodendrocytes in a demyelination model [11]. Recently, we showed that the peptide can target newborn and adult rat NSCs (rNSCs), and modify rNSCs properties *in vitro*, in particular differentiation [12].

To determine the feasibility of using this approach as a therapeutic strategy, it is crucial to obtain the proof of concept that the NFL-TBS.40-63 peptide can also be internalized by human NSCs (hNSCs), and evaluate possible effects in hNSCs after uptake. Here, we investigated the *in vitro* internalization of the NFL-TBS.40-63 peptide in hNSCs isolated from human fetuses and potential effects on their properties. We showed that the NFL-TBS.40-63 peptide enters massively by direct translocation in these cells, without major effect on their viability at low concentration (less than 40 µm). At higher concentrations, the peptide inhibits proliferation and the ability to form neurospheres. These results are consistent with an increase in cell adhesion and their more advanced differentiation state (in the neuronal and astrocytic pathways). To our knowledge, this is the first report to show that a peptide can enter into hNSCs, leading to modified stem cell properties including differentiation. This provides a promising tool to target such cells during regenerative therapy.

## Materials & Methods

### Ethics statement

Human fetuses were obtained after legal abortion with written informed consent from the patient. The procedure for the procurement and use of human fetal central nervous system tissue was approved and monitored by the “Comité Consultatif de Protection des Personnes dans la Recherche Biomédicale” of Henri Mondor Hospital, France. The cells are declared at the “Centre des Ressources Biologiques”of the University Hospital in Angers with reference numbers at the Research Ministry: declaration N° DC-2011-1467; authorization N° AC-2012-1507.

### Cell culture and materials

The hNSCs used in this study were prepared from the central nervous system of first trimester human fetuses, as previously described [13]. Briefly, the cortex was dissected and cut into 1-mm^3^ tissue pieces. After mechanical dissociation, single-cell suspensions were cultured in DMEM/Ham’s F12 culture medium in a 3:1 mixture (Dulbecco’s modified Eagle medium with L-glutamine (DMEM; Lonza, Levallois-Perret, France) and Ham’s F12 (Lonza) supplemented with 1X B27 (Invitrogen Life Technologies, Saint Aubin, France), Epidermal Growth Factor (EGF) (20 ng/ml; R&D systems), basic Fibroblast Growth Factor (bFGF) at 20 ng/ml (R&D systems, Minneapolis, MN 55413, USA), Heparin (5 µg/ml, Merck Millipore, île-de-France, France), 100 U Penicillin, and 1,000 U Streptomycin (Sigma, Saint-Quentin Fallavier, France)). This cell suspension generated proliferating clones containing hNSCs in floating spheres (termed neurospheres). Cells were further expanded and maintained in suspension as neurospheres in uncoated tissue culture dishes and the medium was changed twice a week. Cells were maintained at 37°C in a humidified atmosphere containing 5% CO_2_. The conditioned medium was composed by DMEM/F12 (1:1) (Lonza) added by 1X B27 and 1X 100 U Penicillin, and 1,000 U Streptomycin. This medium induced cell adhesion and differentiation of hNSCs after 10 days in culture as described elsewhere [14, 15].

Peptides were synthetized by Genecust (Dudelange, Luxembourg), Genosphere (Paris, France) or Polypeptide (Strasbourg, France). The NFL-TBS.40-63 peptide (YSSYSAPVSSSLSVRRSYSSSSGS) was coupled to 6-carboxy-fluorescein (FAM) or biotin, and dissolved in sterile water at 0.5 mmol/L.

Chlorpromazine hydrochloride (50 µmol/L), Nystatine (25 µg/ml), 5(N,N-dimethyl) amiloride hydrochloride (DMA, 1 mmol/L), Sodium azide (10 mmol/L) and D-glucose (6 mmol/L) were obtained from Sigma. Paclitaxel (Life Technologies, Illkirch, France) or colchicine (Sigma) was used as positive control in some techniques.

### Internalization of the peptide in hNSCs

The uptake of the peptide by hNSCs was measured using the flow cytometer technique. Briefly, hNSCs were seeded in 6-well plates and treated with increasing concentrations of FAM- coupled NFL-TBS.40-63 peptide for an hour at 37°C. To analyze the internalization mechanism, cells were pre-incubated at 4°C, with an ATP-depleted buffer (sodium azide at 10 mmol/L and D-glucose at 6 mmol/L) or with a panel of endocytosis inhibitors for 30 minutes before the addition of the peptide at 20 µmol/L. Incubation with the peptide was performed in the same conditions. Cells were recovered, washed once with PBS (Phosphate Buffered Saline), and pelleted (1200 rpm– 5 minutes). Cell dissociation was performed with a 26 gauge needle and incubated with 150 nmol/L calcein violet (Invitrogen) for 20 minutes before analysis with Fluorescence-Activated Cell Sorting or flow cytometry (FACS) using BD FACSCanto II (BD Biosciences, Le pont de Claix, France). A minimum of 10,000 events per sample were analyzed, followed by data analysis with the Flowing Software (version 2.5.1).

To visualize the FAM-labeled peptide in hNSCs, cells were dissociated and seeded for 1 hour on coverslips coated with BD Cell-Tak (3.5 µg/cm²) (Corning, Bedford, United Kingdom). Then, they were incubated for 1 hour at 37°C with 20 or 60 µmol/L of FAM-labeled peptide. Cells were washed twice with PBS and fixed with Paraformaldehyde 4% (PFA) (Fisher Scientific, Illkirch, France) for 20 minutes. After washing, coverslips were mounted with a Prolong Gold antifade solution (Life Technologies). Finally, neurospheres were observed with a confocal microscope (Leica TCS SP8; Leica Biosystems, Nanterre, France).

The peptide was also visualized in hNSCs together with different markers using immunofluorescence. For this purpose, cells were seeded on coverslips coated with BD Cell-Tak as described above, and then incubated 1 hour with FAM-labeled peptide and washed twice. Then, they were fixed with PFA 4%, permeabilized 10 minutes with MTSB (PIPES 50 mmol/L; MgSO_4_ 5 mmol/L and EGTA 5 mmol/L) in PBS and saturated with BSA 4% in PBS. Two washes were performed between each step. Cells were incubated overnight at 4°C with the primary antibody (rabbit anti α-tubulin 1/500 (SAB3501072-Sigma); rabbit anti nestin 1/100 (N5413-sigma); or rabbit anti CD133 1/100 (18470-1-AP-Proteintech (Manchester–United Kingdom)). After washing twice, the cells were incubated with AlexaFluor 568-coupled anti-rabbit antibodies 1/200 (Invitrogen) for 90 minutes in the dark and washed twice. Finally, cells were counterstained with 3 µmol/L 4’6-diaminido-2-phenylindole (DAPI; Sigma) for seven minutes and the coverslips were mounted with Prolong.

### Cell viability and microtubule network disruption

The cell viability was evaluated with the AnnexinV-FITC Kit (Miltenyi, Paris, France) after 72 hours incubation of the cells with NFL-TBS.40-63 peptide or Paclitaxel. In short, cells were seeded in 6-well plates and incubated with increasing concentrations of biotinylated peptide or Paclitaxel (0.1 µmol/L) for 72 hours at 37°C. The cells were collected and centrifuged at 1200 rpm for 10 minutes. Following washing and centrifugation (1200 rpm– 10 minutes), the cells were stained with FITC-Annexin-V for 10 minutes at room temperature in the dark. Then, the cells were washed, dissociated, counterstained with Propidium Iodide (PI) (1 µg/ml—Sigma) and analyzed by FACS. A minimum of 10,000 events per sample were analyzed, followed by data analysis with the Flowing Software (version 2.5.1).

The possible effect of the peptide on the microtubule network was analyzed using immunofluorescence. The cells were first incubated for 24 hours with the FAM-NFL peptide, and then stained with the anti-α-tubulin (1/500 T6199-Sigma). Primary antibody was exposed using the AlexaFluor 568-coupled anti-mouse antibody 1/200 (Invitrogen), which was incubated for 90 minutes in the dark. After washing and counterstaining with DAPI, cells were visualized and analyzed by confocal microscopy. The number of cells with disrupted microtubule network was counted manually.

### Cell proliferation and cell cycle analysis

To analyze the effect on the cell cycle, hNSCs were seeded in 6-well plates and treated for 72 hours with increasing concentrations of biotinylated-NFL-TBS.40-63 peptide, or with colchicine at 1µg/ml. The cells were collected and centrifuged at 1200 rpm for 10 minutes, followed by cells dissociation and permeabilization with Tween 0.1% in PBS. Then, the cells were fixed with 70% ethanol, centrifuged and suspended in 1 mg/ml RNase for 30 minutes at 37°C, and analyzed by FACS after adding 10 µg/ml of PI. Proliferation of hNSCs was determined with two techniques: the Trypan blue exclusion test and the CyQUANT assay kit (Fisher).

For the Trypan Blue exclusion test, neurospheres were seeded in 24-well plates and treated for 72 hours with increasing concentrations of 1 µg/mL biotinylated peptide or colchicine. Neurospheres were dissociated with Accumax enzyme, and the number of cells was counted following Trypan blue (Sigma, dilution 1:2) staining. At least three wells per condition were analyzed.

For the CyQUANT assay tests, the cells were dissociated with Accumax enzyme, seeded and treated with colchicine (1 µg/L) or increasing concentrations of NFL-TBS.40-63 peptide in black 96-well plates. After 72 hours, supernatants were transferred on black 96-well plates coated with BD Cell-Tak (3.5 µg/cm²), and the cells were allowed to adhere for 1 hour. Then, wells were washed twice with PBS and frozen at -80°C for at least 24 hours. CyQUANT GR dye buffer (200 µl) was added per well, and the fluorescence was measured with a fluorescence microplate reader (Spectra max M2; Molecular Devices, Sunnyvale, USA) (excitation: 480 nm; emission: 520 nm). All experiments were performed at least three times.

The number of cells in the replicative state was determined using BrdU fixation (colorimetric technic) or EdU (FACS) labeling. FACS was performed using the Click-iT Plus EdU Flow Cytometry Assay Kits (Fisher) following 72 hours incubation of increasing concentrations of NFL peptide or with 1 µg/ml of colchicine. Briefly, after 72 hours treatment, cells were incubated with 10 µmol/l of EdU for five hours. Cells were harvested and washed with 1% BSA in PBS by centrifugation at 800g for 10 minutes. The Cells were fixed and permeabilized, with washing between all stages. The cells were then incubated with the detect EdU, washed and analyzed by FACS. A minimum of 10,000 events per sample were analyzed, followed by data analysis with the Flowing Software (version 2.5.1).

BrdU labeling was evaluated using the BrdU cell proliferation ELISA kit (Abcam, Cambridge, UK). Briefly, cells were incubated with 1 µg/ml of colchicine or increasing concentrations of NFL peptide for 72 hours on a BD Cell-Tak coated 96-well plates. Incubation of BrdU was performed for five hours. Then, the cells were fixed and their DNA was denatured for 30 minutes with the fixative solution. The cells were washed and incubated with the anti-BrdU antibody for 1 hour. BrdU was exposed using the HRP conjugated antibody and its TMB substrate. Positive BrDU cells were analyzed by colorimetry (absorbance reading at 450 nm) with a fluorescence microplate reader.

### Neurosphere formation ability

Neurosphere formation was evaluated by counting neurospheres after 7 days in the presence of the biotinylated-peptide. Briefly, the cells were dissociated with Accumax enzyme and seeded in 24-well plates, with each well divided in five squares. The cells were treated with increasing concentrations of peptide or colchicine (1 µg/ml) for 7 days, and the culture medium changed after 4 days. Then, images of each well were captured five times with a Power Stat G15 digital camera. The neurosphere number and size were measured with the ImageJ software (NIH, USA). The number of neurospheres was reported by cm² of surface. The experiments were repeated at least three times.

### Cell adhesion

To measure the number of cells that had adhered, the CyQUANT cell Proliferation Assay Kit (Molecular Probes, Oregon, US) was used. Briefly, cells were dissociated and seeded in black 96-well plates (10,000 cells/well). hNSCs were treated with increasing concentrations of peptide for 72 hours. Then, the wells were washed twice with PBS and frozen at -80°C for at least 24 hours. CyQUANT GR dye buffer (200 µl) were added to each well, and the fluorescence was measured with a fluorescence microplate reader (Spectra max M2) (excitation: 480 nm; emission: 520 nm). All experiments were performed at least three times.

The cell adhesion was also investigated by immunofluorescence. Briefly, the cells were treated with increasing concentrations of biotinylated peptide on coverslips without coating. After 72 hours, all supernatants were transferred onto coverslips coated with BD Cell-Tak to allow adhesion of cells. All coverslips were fixed with PFA 4%, washed and incubated for 10 minutes with permeabilized buffer. After washing with PBS, the cells were stained with Phalloïdine TRITC (P1951-Sigma) for 1 hour, washed and counterstained with DAPI.

### Cell differentiation

To analyze the effect of the peptide on cell differentiation, hNSCs were seeded in 6-well plates and treated with 20 or 60 µmol/L of biotinylated peptide for five days. The cells were collected and centrifuged at 1200 rpm for 10 minutes, followed by dissociation for 10 minutes at 37°C with Accumax enzyme (Stemcell Technologies, Grenoble, France) and centrifugation at 2000 rpm for 10 minutes. The cells were incubated for 10 minutes in the dark with buffer (0.5% BSA / 2 mmol/L EDTA in PBS) containing A_2_B_5_-PE (10 µg/ml) (Miltenyi Biotec, Auburn, USA) at or CD90-PerCP-Cy5.5 (0.4 µg/ml) (Miltenyi Biotec) or at 4°C in the dark for 10 minutes with buffer (0.5% BSA in PBS) containing O4-PE at 1/100 (Miltenyi Biotec). One milliliter buffer was added before centrifugation at 2000 rpm for 10 minutes. The cells were stained with calcein violet and analyzed by FACS after 20 minutes incubation. A minimum of 10,000 events by sample were analysed, followed by data analysis with the Flowing Software (version 2.5.1). The graphics are performed with FCS express 6 Flow Cytometry software.

To study if the peptide loses efficiency in a conditioned medium that induces cell differentiation, the cells were incubated in conditioned medium with or without peptide for 10 days. Briefly, 6-well plates were coated with poly-D-lysine (100µg/ml Sigma) for 1 hour at 37°C before seeding hNSCs in the conditioned medium alone (control), or containing 20 µmol/l NFL peptide or 60 µmol/l NFL peptide. The medium was changed every 4–5 days until experimentation. The same protocol was used to evaluate differentiation by FACS with CD90, A_2_B_5_ and O4.

Cell differentiation was also investigated by RT-PCR (Reverse Transcription polymerase chain reaction) with different probes from the four nervous system lineages (stem cell, neuron, astrocyte and oligodendrocyte), after five days in the presence of increasing concentrations of the peptide. All probes came from the “Plateforme d’Analyses Cellulaire et Moléculaire”(PACEM), University of Angers (France), as detailed in Table 1. Briefly, cells were seeded in 6-well plates and treated for five days with 20 or 60 µmol/L of NFL biotinylated peptide. The cells were washed with PBS and frozen as dry pellet before RNA extraction and purification. The total RNA extraction was performed with the RNeasy Micro kit (Qiagen, Courtaboeuf, France) and the total extract was recovered in 14 µl RNase-free H_2_O. The reverse transcription in cDNA was done with the Reverse Transcriptase SuperScript II Kit (Invitrogen). The purification of cDNA was done with the QIAquick PCR Purification Kit (Qiagen). In total, 40 µl purified cDNA were recovered. Quantitative RT-PCR was achieved with the Maxima SYBR Green qPCR master mix (Fermentas/ThermoFisher Scientific). The amount of cDNA was normalized with the glyceraldehyde-3-phosphate dehydrogenase (GAPDH), and the relative gene expression was compared to the control condition using the 2^-ΔΔCt^ method [16].

**Table 1:** Primer sequences used for reverse transcription-polymerase chain reaction.

Gene	Full name	NM accession	Sequence
**CD133**	Prominin 1	NM 006017.2	For 5’CTCAGACTGGTAAATCCCCC
			Rev 5’GACGCTTTGGTATAGAGTGC
**CD44**	CD44 molecule	NM 001202557.1	For 5’GATCCTCCAGCTCCTTTCG
			Rev 5’GGAATACACCTGCAAAGCG
**CNP**	2’,3’-cyclic nucleotide 3’phosphodiesterase	NM 033133.4	For 5’CCAAGTTTTGTGACTACGGG
			Rev 5’AGCTTGTCCACATCACTCG
**DCX**	Doublecortin	NM 001195553.1	For 5’GTACGTTTCTACCGCAATGG
		NM 000555.3	Rev 5’ATCCATGCTTCCGATCTTCC
		NM 178153.2	
		NM 178152.2	
		NM 178151.2	
**GALC**	Galactosylceramidase	NM 000153.3	For 5’AGCATCACTTCACGCTACG
			Rev 5’CTTCCTGCAATGAACACACC
**GAPDH**	Glyceraldehyde-3-phosphate dehydrogenase	NM 001256799.1	For 5’-CAAAAGGGTCATCATCTCTGC
		NM 002046.4	Rev 5’ AGTTGTCATGGATGACCTTGG
**GFAP**	Glial fibrillary acidic protein	NM 002055.4	For 5’CTACAGGAAGCTGCTAGAGG
			Rev 5’TTAATGACCTCTCCATCCCG
**GLAST**	Solute carrier family 1	NM 001166696.2	For 5’TGCAAAGAAGAGACCCTCC
	(glial high affinity glutamate transporter)		Rev 5’CTGTGAGCAGCACAAAAGC
**NCAM**	Neural cell adhesion molecule 1	NM 001242608.1	For 5’TACAAAGCTGAGTGGAGAGC
		NM 001242607.1	Rev 5’CGTCTTGAACTCGGAGGC
		NM 001076682.3	
		NM 181351.4	
		NM 000615.6	
**NESTIN**	Nestin	NM 006617.1	For 5’AGAAACAGGGCCTACAGAG
			Rev 5’AAAGCTGAGGGAAGTCTTG
**SOX2**	SRY-box2	NM 003106.3	For 5’GCAGTACAACTCCATGACCA
			Rev 5’GATCATGTCCCGGAGGTC
**TUBBIII**	Tubulin, beta 3 class III	NM 006086.3	For 5’CCAGTATGAGGGAGATCG
			Rev 5’CACGTACTTGTGAGAAGAGG

Accession numbers correspond to the transcripts for the gene. When several transcripts for the same gene exist, the probes were designed to amplify all transcripts.

### Statistical analysis

All experiments were repeated at least three times. Results were presented as mean ± standard error of the mean (SEM). Multiple group comparisons were performed with Tukey’s Honest significant Difference test or Sidak using Prism version 7.00 (GraphPad PRISM software). Asterisks indicate significantly different results: * P≤0.05; ** P≤0.01; *** P≤0.001; ****P≤0.0001.

## Results

We first investigated the uptake of the neurofilament-derived peptide NFL-TBS.40-63 in hNSCs and its internalization mechanism. Then, we analyzed the effects of this peptide on cell viability and hNSC properties.

### Uptake of NFL-TBS.40-63 peptide by hNSCs occurs by a passive direct translocation

**Fig 1:**
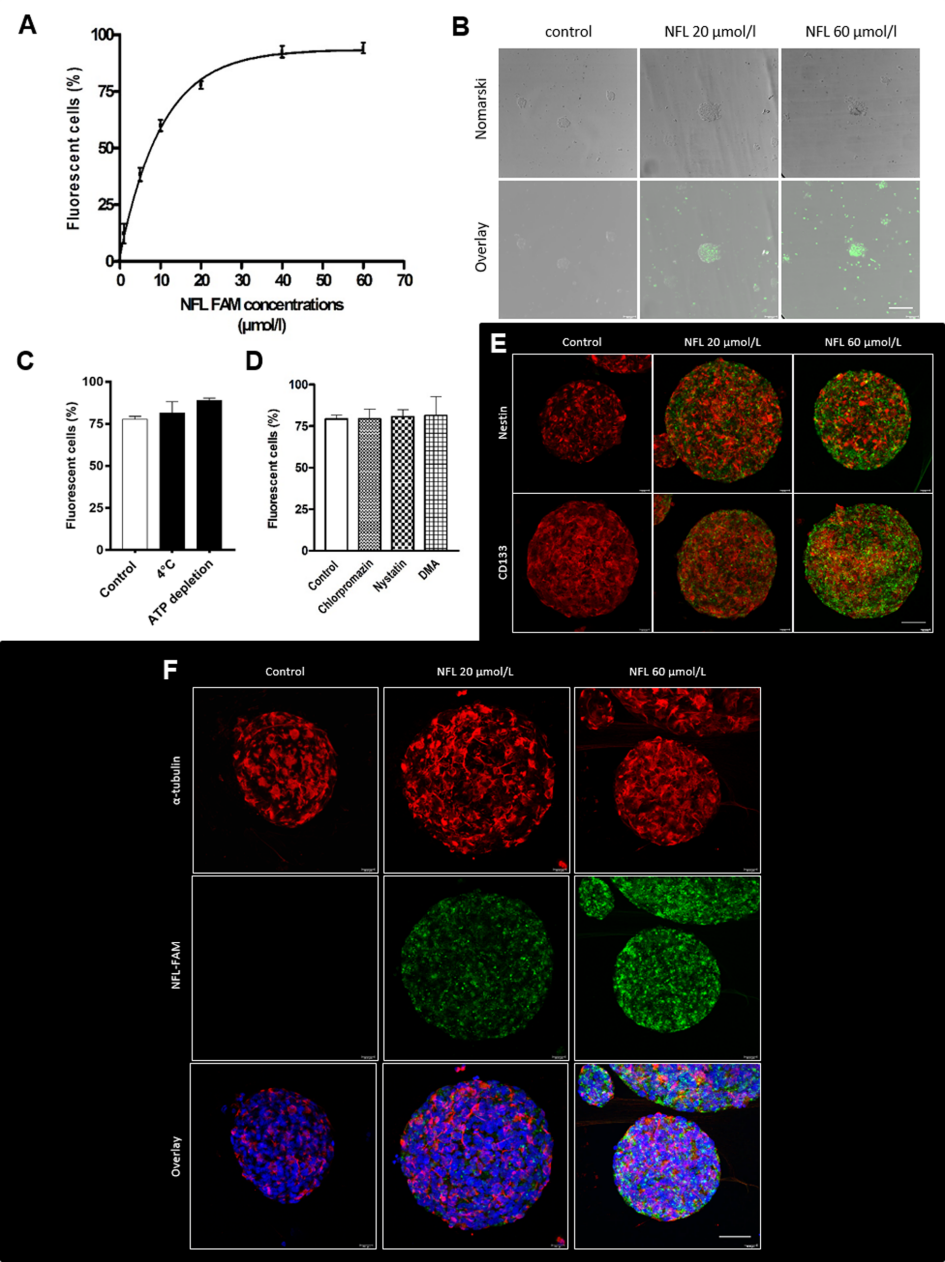
Uptake of the FAM-labeled NFL-TBS.40-63 peptide in hNSCs. **(A)** Percentage of FAM-labeled NFL-TBS.40-63 internalized by hNSCs with increasing concentrations of peptide after 1 hour incubation at 37°C. **(B)** Visualization of the FAM-labeled peptide after 1 hour incubation with 20 or 60 µmol/L of peptide. Scale bar: 50 µm. Green: FAM-labeled peptide **(C)** Internalization of 20 µmol/L of peptide after pre-treatment at 4°C or ATP depletion or **(D)** in the presence of inhibitors of the endocytosis pathways. Data are presented as means ± SEM. *P<0.05. **(E-F)** Immunofluorescence after incubation of cells for 1 hour with 20 or 60 µmol/L FAM-labeled peptide. Cells were stained with specific neural stem cells markers: Nestin and CD133 **(E)** or with α-tubulin **(F)**. Images were taken with the confocal microscope. Red: Nestin, CD133 or α-tubulin; Green: FAM-labeled peptide; Blue: DAPI. Scale bar: 50 µm.

Cells were incubated for 1 hour with increasing concentrations of FAM-labeled NFL-TBS.40-63 peptide, and the percentage of labeled-cells was analyzed by flow cytometry (Fig 1A). At 5 µmol/L, the peptide was internalized by 38.38 ± 2.90% of cells, whereas at 20 µmol/L it was internalized by 76.34 ± 1.62% of cells. A maximum was reached for 40 µmol/L of peptide. These results were confirmed by microscopy after incubating the cells for 1 hour with the FAM-peptide and visualization by confocal microscopy (Fig 1B).

The influence of low temperature, ATP depletion and endocytosis inhibitors were analyzed to assess whether internalization depends on endocytosis pathways. Unlike endocytosis pathways, direct translocation is an energy and temperature independent pathway. Thus, the direct translocation mechanism of penetration can be shown by reducing the temperature to 4°C or depleting ATP (source of energy) [17].

First, we studied the temperature and energy dependent pathways by pre-treatment of NSCs for 30 minutes at 4°C or with a buffer containing 10 mmol/L sodium azide and 6 mmol/L 2-deoxy-D-glucose (ATP-depleted buffer), before adding the NFL peptide (20 µmol/L) in the same conditions for 1 hour. Neither the low temperature nor the ATP depletion condition modified peptide internalization (81.29 ± 6.89% and 88.76 ± 1.53%, respectively, compared with 77.80 ± 1.70% in control; Fig 1C). These results indicate that peptide uptake by hNSCs occurs via a direct translocation passive mechanism. To really exclude the active pathways, cells were pre-incubated with a complete panel of inhibitors against the three principal endocytosis pathways: chlorpromazine, nystatin and DMA [18]. Chlorpromazine inhibits clathrin-dependent endocytosis by reversible translocation of clathrin and its adapter proteins from the membrane to intracellular vesicles [19]. Nystatin inhibits caveolin-dependent endocytosis by depletion of cholesterol from caveolae [20], while DMA is known to inhibit micropinocytosis by inhibition of the Na^+^/H^+^ exchange. The presence of these inhibitors did not prevent peptide internalization (Fig 1D), which still stained approximately 80% of the cells. These data confirmed that uptake of NFL peptide in hNSCs occurs via a passive pathway. The peptide can be visualized by immunofluorescence thanks to fluorescein, and cells were also stained with α-tubulin (Fig 1F) or with specialized-NSCs markers: Nestin and CD133 (Fig 1E). All the raw data supporting these results are available (S1 File).

### The NFL-TBS.40-63 peptide has no effect on cell viability and microtubule network

**Fig 2:**
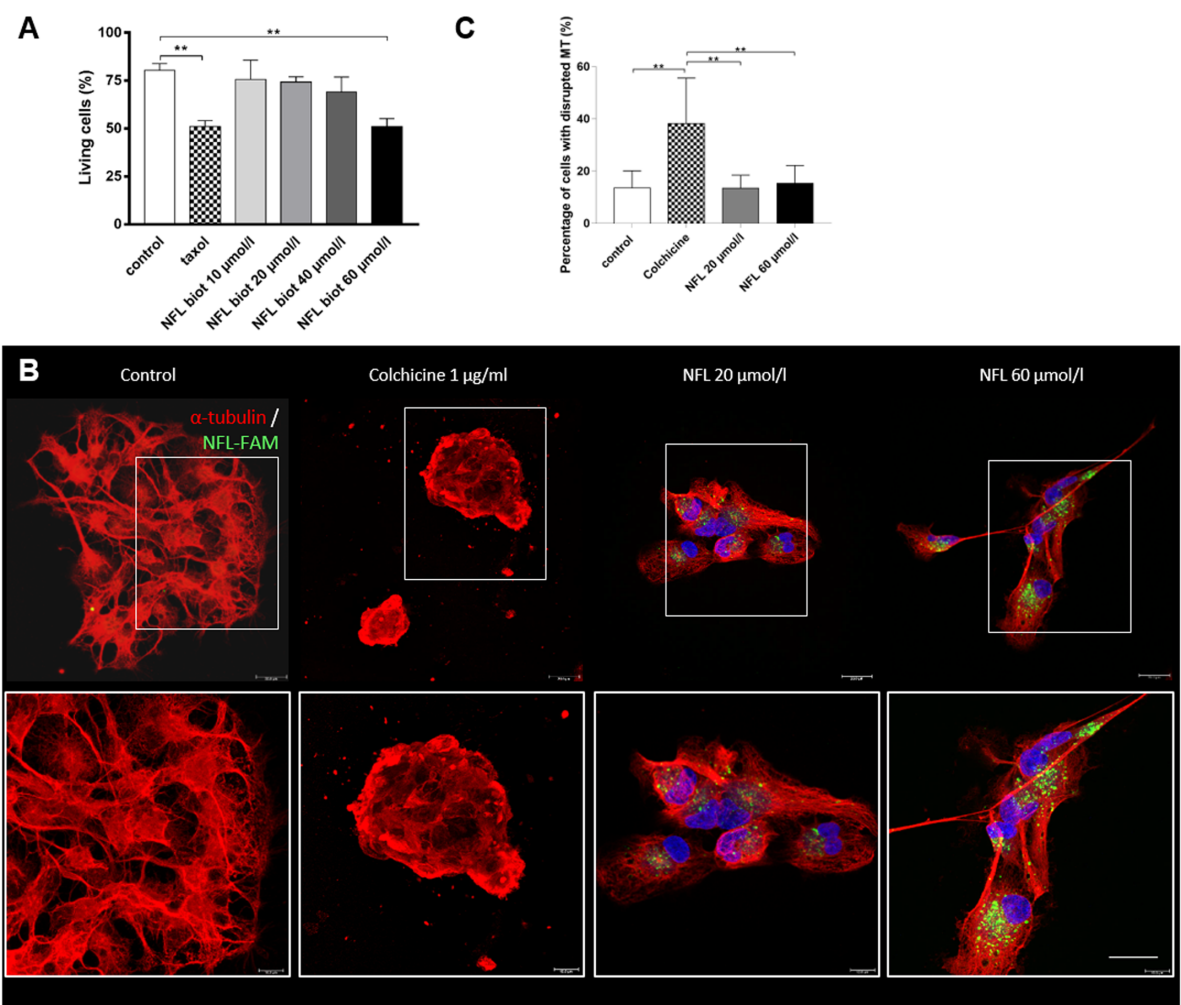
The NFL-TBS.40-63 peptide has no toxic effect on cell viability and the microtubule network. **(A)** Percentage of viable cells was evaluated after incubation for 72 hours with increasing concentrations of NFL-TBS.40-63 peptide or with Taxol (0.1 µmol/L). Cell viability was measured by FACS (Annexin-V-FITC / PI assay kit). **(B)** Effect of 20 or 60 µmol/L of FAM peptide (green) on the microtubule network (α-tubulin: red; NFL FAM peptide: green) after 24 hours. Images were taken with the confocal microscope. Scale bar: 20 µm. **(C)** Percentage of cells with a disrupted microtubule network in control condition, or with different treatments (colchicine, NFL 20 or 60 µmol/l). Data presented as means ± SEM. **P<0.01.

Paclitaxel (Taxol) (for the cell viability) or colchicine (for all other experiments) were used as positive controls. To study potential effects of the peptide on hNSCs, we evaluated the viability of hNSCs after 72 hours incubation with the NFL peptide, at different concentrations, using the flow cytometry technique with the Annexin-V-FITC/PI assay kit. Fig 2A shows the percentage of viable cells (control: 80.31 ± 3.585%). Taxol at 0.1 µmol/L was used as a positive control to reduce viability (50.92 ± 3.122%). The NFL peptide has no major effect on the viability of cells up to 40 µmol/L (74.10 ± 2.888 at 20 µmol/L and 68.99 ± 7.922 at 40 µmol/L). At 60 µmol/L, viability was reduced to 50.94 ± 4.207%. These results show that the NFL-TBS.40-63 peptide has no major effect on the viability of hNSCs below 40 µmol/L (Figs 2A and S1A). All the raw data supporting these results are available (S1 File).

Then, we investigated the effect on the microtubule network by incubating hNSCs with increasing amounts of peptide (20 or 60 µmol/L) for 24 hours. Fig 2B and 2C show the microtubule network of cells stained with an anti-α-tubulin antibody and their quantification. The microscopy visualization showed that no major difference was observed between control cells and those treated with the NFL peptide, unlike with colchicine where there are fewer extensions and an altered microtubule network (Fig 2B). Quantification confirmed that only the colchicine treatment was able to alter the microtubule network (13.67 ± 2.455% of cells with disrupted MT network compared with 38.29 ± 7.781%, control condition and colchicine respectively. 13.57 ± 1.853 and 15.40 ± 2.789% corresponding to NFL 20 and 60 µmol/l, respectively; Fig 2C). These results demonstrate that the peptide had no major effect on the microtubule network.

### Effects of the NFL-TBS.40-63 peptide on the proliferative properties of hNSCs

**Fig 3:**
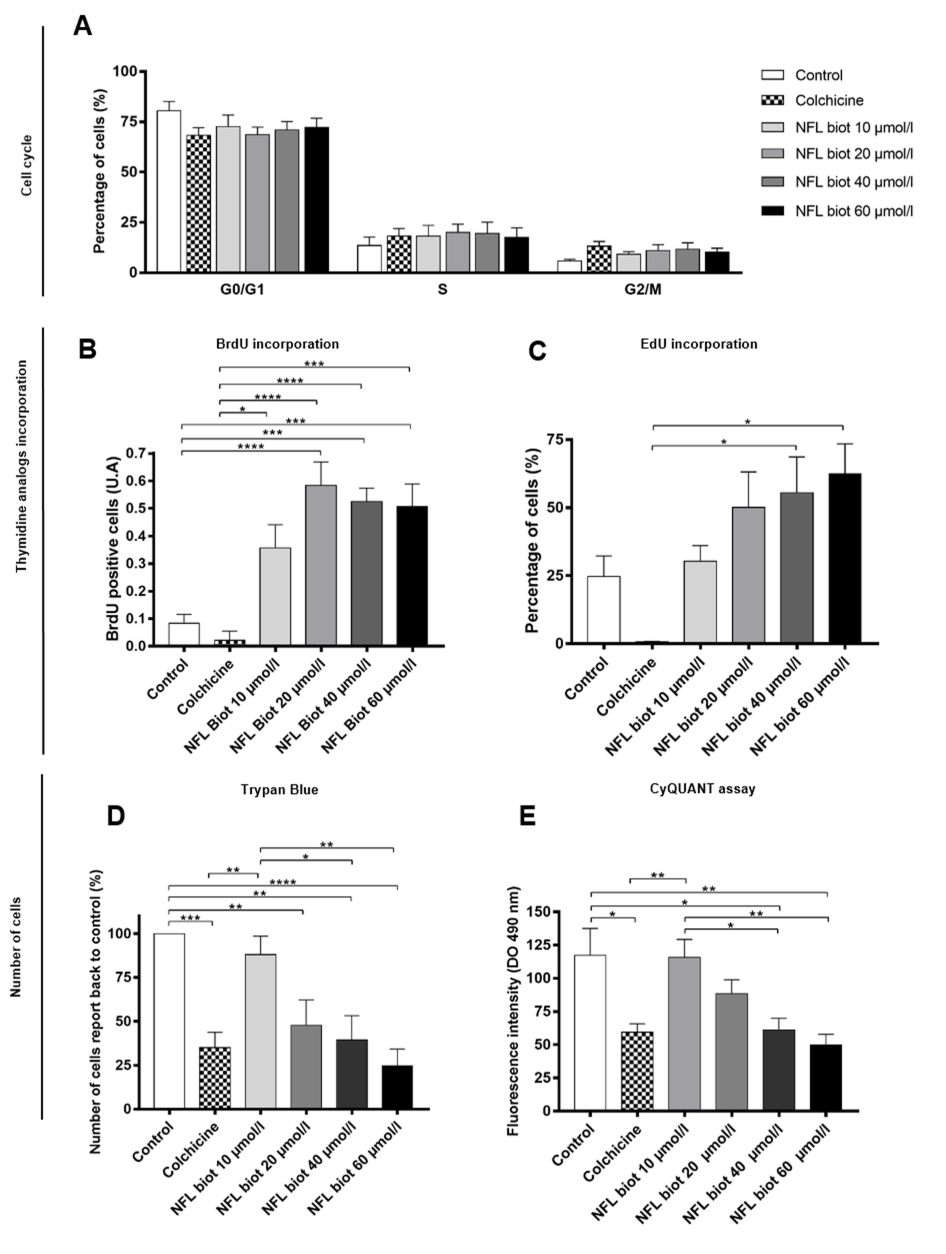
The NFL-TBS.40-63 peptide affects the proliferation of hNSCs. **(A)** Percentage of hNSCs at G0/G1, S or G2/M of the cell cycle after treatment with colchicine (1µg/ml) or increasing concentrations of biotinylated-NFL-TBS.40-63 peptide. Data are presented as means ± SEM. **(B-C)** Thymidine analogue incorporation: The number of BrdU **(B)** or EdU positive cells **(C)** was counted after 72 hours incubation with 1 µg/ml of colchicine or with increasing concentrations of peptide, and compared to the control condition. Thymidine analogues were incubated for 5 hours before the end of the test. Data presented as means ± SEM. **(D-E)** Cell proliferation: Number of cells after 72 hours incubation with the NFL peptide was quantified using Trypan blue exclusion **(D)** or using the CyQUANT assay **(E)**. *P<0.05, **P<0.01, ***P<0.001, ****P<0,0001.

We analyzed the effect of increasing concentrations of the peptide for 72 hours on the cell cycle (Fig 3A). We used colchicine as a positive control to block cells in the G_2_/M phase [21] (13.12 ± 1.97% against 5.9 ± 0.76%, in respectively colchicine or control conditions). The cell cycle of hNSCs was not significantly modified by the NFL-TBS.40-63 peptide (G_0_/G1 phase: 80.53±4.62% vs 68.5±3.84%, S phase: 13.57±4.14% vs 20.07±4.04%, G_2_/M phase: 5.9±0.76% vs 11.03±2.7%, condition control and NFL-TBS.40-63 at 20 µmol/L, respectively; Fig 3A). However, results point towards a decrease of G0/G1 phase and an increase of the other two cell cycle phases. To further explore this effect, we also analyzed the incorporation of thymidine analogues (BrdU and EdU– 5 hours incubation) after 72 hours treatment with the NFL peptide. We observed that the integration of both analogues seemed to be increased with increased concentrations of the peptide, while the incorporation was reduced in the presence of colchicine (Figs 3B, 3C and S1B). Indeed, EdU incorporation increased from 24.7 ± 7.605% in control to 50.11 ± 13.04 or 62.38 ±11.11 with respectively 20 or 60 µmol/l of peptide, and decreased at 0.65 ± 0.2723 with colchicine.

Next, we determined if proliferation could be affected by the presence of the peptide. To that end, we quantified the cell number after 72 hours incubation using two techniques. The first approach used cell quantification with Trypan blue exclusion (Fig 3D). The number of cells in each experiment was compared to the number of cells in the control condition. The presence of colchicine reduced cell number by about 75% when compared to control (35.09 ± 8.68%). When cells were incubated with increasing concentrations of peptide, cell number was significantly reduced in a dose-dependent manner (Fig 3D; 20 µmol/L: 47.68 ± 14.59% and 60 µmol/L: 24.48 ± 9.79%). Similar results were obtained using the second method, where nuclei were stained with a fluorescent probe (CyQUANT GR) (Fig 3E). The number of cells after 72 hours was gradually decreased when hNSCs were incubated with increasing concentrations of peptide, thus confirming the anti-proliferative effect of the peptide on hNSCs (117.2 ± 20.38 AU in control conditions compared with 88.53 ± 10.39 AU at 20 µmol/L; 61.28 ± 8.607 AU at 40 µmol/L and 49.86 ± 8.023 AU at 60 µmol/L).

### Effect on neurosphere formation

**Fig 4:**
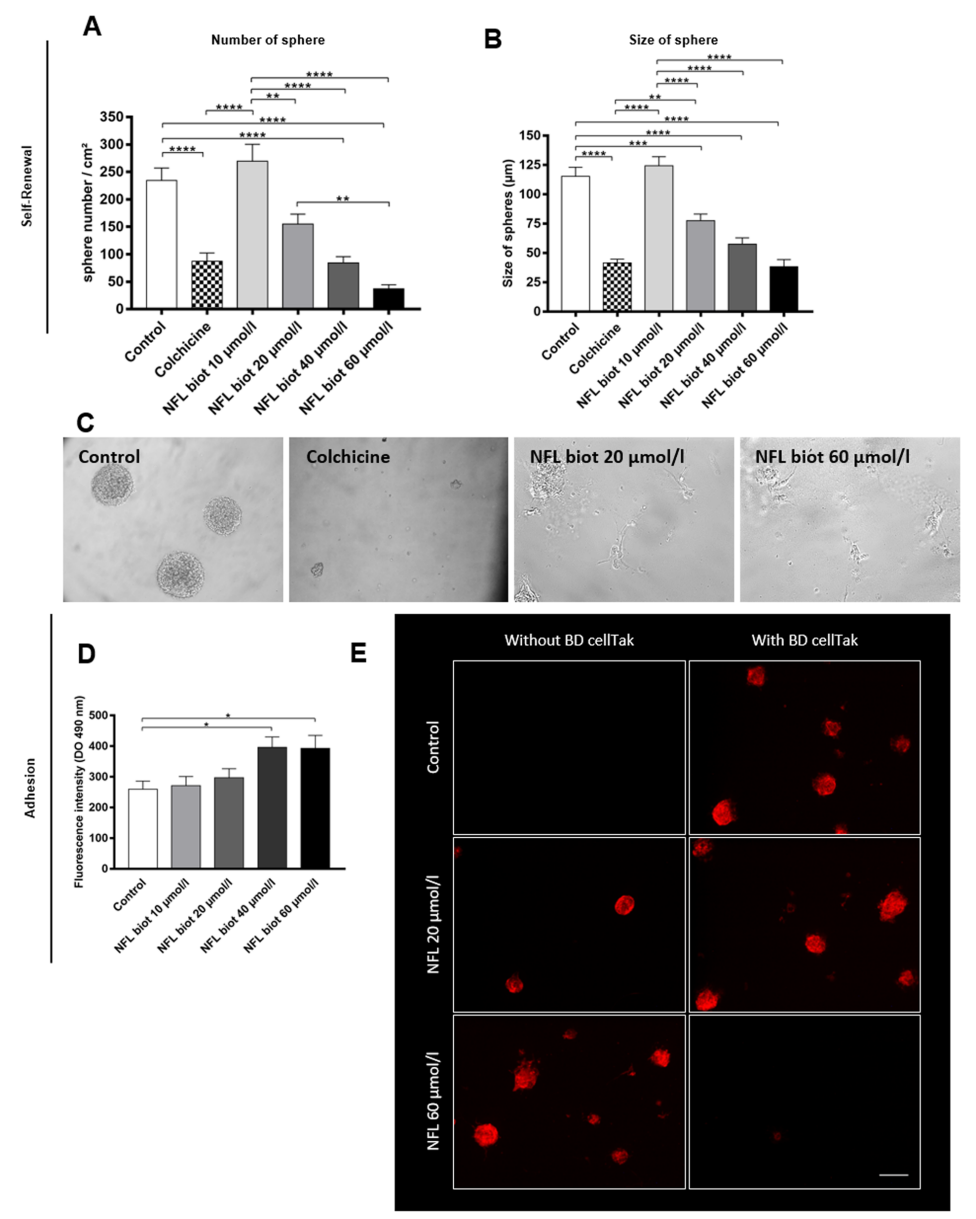
The NFL-TBS.40-63 peptide affects the capability to form neurospheres and increase adhesion of hNSCs. **(A-C)** Sphere formation from hNSCs: quantification of the number of neurospheres per square centimeter **(A)** and their size **(B)** after 7 days incubation with increasing concentrations of the peptide, or with 1 µg/ml of colchicine. Scale bar: 150 µm. **(C)** Morphology of neurospheres observed after 7 days incubation in normal conditions, or with colchicine, or with the NFL peptide at 20 or 60 µmol/L. **(D-E)** Quantification of adherent cells after 72 hours with increasing concentrations of NFL peptide using the CyQUANT assay **(D)** or by immunostaining of cells after 72 hours with increasing concentrations of biotinylated peptide **(E)**. Adherent cells were observed on coverslips without coating (top pictures), while non-adherent (floating) cells were recovered following centrifugation and 1 hour incubation on BD Cell-Tak coated coverslips (Bottom pictures). Cells were visualized by Phalloïdin staining (red) and DAPI staining the nucleus (blue). Scale bar: 100 µm. Data presented as means ± SEM. *P<0.05, **P<0.01, ***P<0.001, ****P<0,0001.

Then, we evaluated the ability of hNSCs to create new neurospheres. Each dissociated cell can create a clone of itself in proliferative medium to form a neurosphere. After 7 days of culture in the presence of increasing concentrations of peptide or colchicine (1 µg/ml), we quantified the number and the size of spheres. Fig 4A represents the average number of spheres per square centimeter after 7 days of culture. The number of neurospheres was significantly decreased with 20 µmol/L of NFL-TBS.40-63 peptide, as well as in the presence of 60 µmol/L of peptide. Moreover, the size of the neurospheres was also reduced in the presence of the peptide (Fig 4B). Both number and size of neurospheres after 7 days of culture with 1 µg/ml of colchicine were reduced by 60%. Fig 4C represents typical neurospheres after 7 days in control conditions, or in the presence of colchicine, or with 20 and 60 µmol/L of peptide. In control conditions, the neurospheres were round, while only individual cells were found in the presence of colchicine. The presence of 20 µmol/L or 60 µmol/L of peptide induced an increased number of adhesive cells, which lost the round shape typical of neurospheres (Fig 4C).

We also quantified the number of cells that were still adhering with the CyQUANT assay kit. The number of adherent cells proportionally increased with the increased concentration of the peptide. We noticed a significant increase of cell adhesion with 40 µmol/L of peptide (from 259.8 ± 25.11 in control conditions to 396.1 ± 31.49 with 40 µmol/L of NFL peptide; Fig 4D). We further confirmed these results using fluorescence staining on coverslips without any coating. Fig 4E (left panels) shows that an increased number of cells was systematically observed in the presence of 60 µmol/L of peptide, and when compared to the absence of peptide. To be sure that the same number of cells was seeded on coverslips, all supernatants were transferred on BD Cell-Tak coated coverslips (Fig 4E, right panels), and then incubated for 1 hour before being stained. Thus, neurospheres that were floating in the control condition (without peptide) were all observed because they adhere on BD Cell-Tak coverslips. In contrast, no neurospheres were floating in the presence of 60 µmol/L of peptide, because no neurospheres were observed when the supernatant was transferred on BD Cell-Tak coated coverslips. No major difference was observed between the control (no peptide) and 20 µmol/L of peptide, as shown after quantification with the CyQUANT assay kit (259.8 ± 25.11 UA compared with 296,8 ± 28.34 UA in controls or with 20 µmol/L, respectively). Taken together, these data indicate that the peptide induces increased cell adhesion. Since the increased adherence of cells can be correlated with an increased number of differentiating cells, we next evaluated the effect of the peptide on the differentiation process.

### Effect on hNSC differentiation

**Fig 5:**
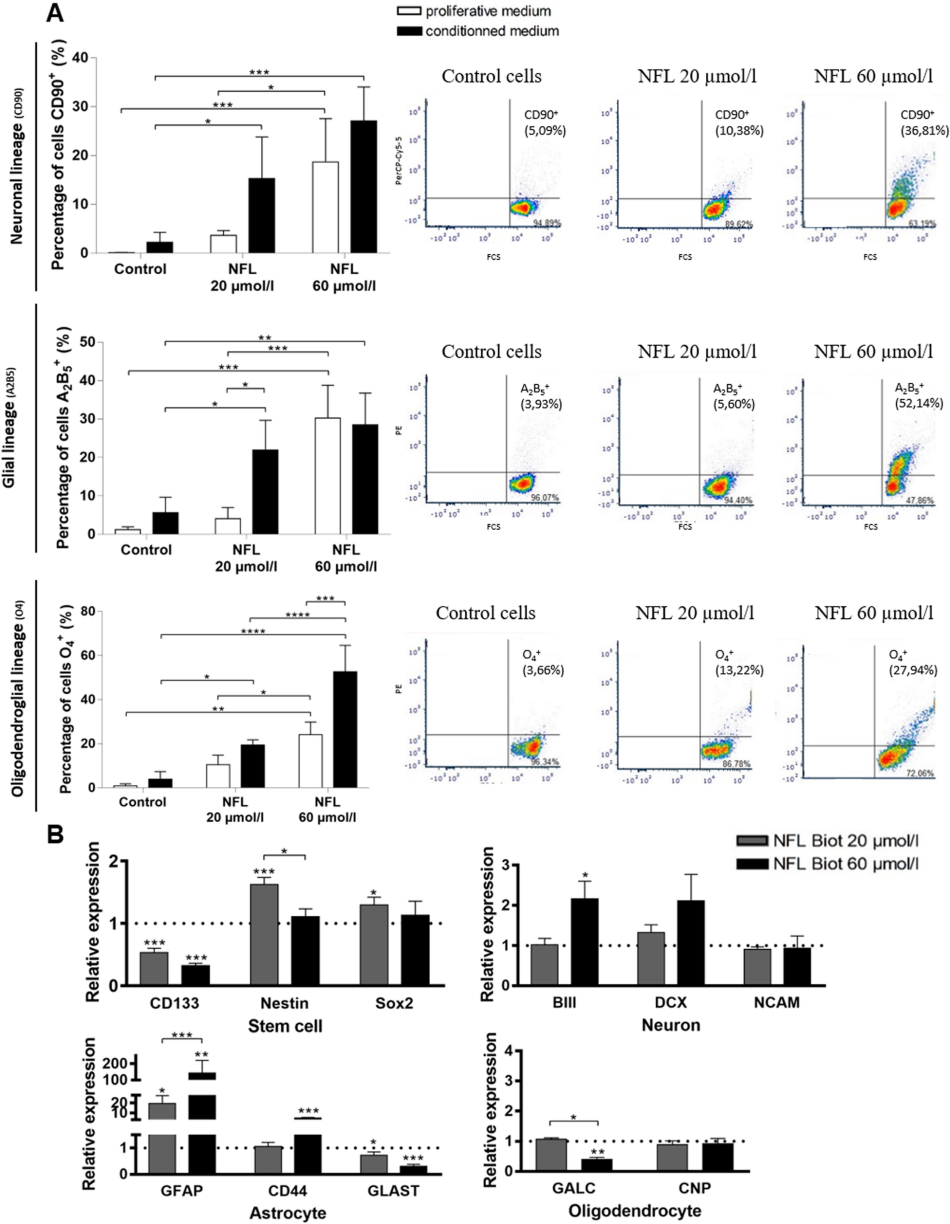
The NFL-TBS.40-63 peptide increases differentiation of hNSCs. **(A-B)** Differentiation of cells induced after 5 days by increasing concentrations of NFL peptide in a proliferative or conditioned medium. **(A)** Percentage of cells which express the neuronal marker CD90 (top graph), the glial marker A2B5 (medium graph) or the oligodendroglial marker O4 (bottom graph) after 5 days in the presence of the NFL peptide at 20 or 60 µmol/L in the proliferative medium (white bars) or the conditioned medium (dark bar). Markers were analyzed by FACS technique and some examples are given in the left part of each graph. **(B)** The relative expression level of *CD133*, *Nestin*, *Sox2*, *βIII tubulin*, *DCX*, *NCAM*, *GFAP*, *CD44*, *GLAST*, *GALC*, *CNP* genes was quantified by reverse transcription polymerase chain reaction after 5 days with 0 (control condition), 20 or 60 µmol/L of peptide. The relative gene expression was compared with control conditions after normalization with the *GAPDH* gene (value of the control condition = 1) with the 2^-ΔΔCt^ method. All data were presented as means ± SEM. *P<0.05, **P<0.01, ***P<0.001, ****P<0,0001. Stars above bars represent significant data compared to control.

We first investigated the neural/neuronal or glial differentiation of hNSCs by quantifying the glial or neuronal progenitor markers (A2B5 and CD90, respectively) and the pre-oligodendrocyte marker (O4), by FACS technique after 5 days in culture in a proliferative medium containing 20 or 60 µmol/L of peptide. We show that all the markers were increased by the presence of peptide (Fig 5A) in a proliferative medium (CD90: 0.0825 ± 3.663 in controls vs 3.663 ± 0.556 at 20 µmol/L and 18.65 ± 3.983 at 60 µmol/L; A2B5: 1.193 ± 0.4333 in controls vs 4.037 ± 1.687 at 20 µmol/L and 30.21 ± 4.939 at 60 µmol/L; O4: 0.847 ± 0.5733 in controls vs 10.47 ± 2.504 at 20 µmol/L and 24.07 ± 3.344 at 60 µmol/L). These results indicate that cells move towards a more differentiated state after 5 days in the presence of the peptide. No specific pathway seems to be induced by the peptide since the three markers were similarly increased.

We also determined whether the fate of hNSCs in a conditioned medium could be affected by the presence of the peptide. We observed that in conditioned medium (control condition), cells express more markers of differentiation than in proliferative medium (CD90: 2.22 ± 1.007; A2B5: 5.593 ± 2.334; O4: 3.953 ± 1.961 in conditioned medium). Moreover, the presence of peptide does not seem to induce a specific differentiating pathway when cells are incubated in the conditioned versus the proliferative medium. However, the differentiation pathway was boosted by the peptide in the conditioned medium (for both peptide concentrations). The conditioned medium stimulates the differentiation of cells in the three pathways, and the peptide over-stimulates this differentiation, but without increasing a selective pathway.

To better characterize the differentiation of hNSCs after 5 days in the presence of the NFL-peptide, a RT-qPCR screening was performed using different markers from the four differentiation pathways (stem cell, neuron, astrocyte or oligodendrocyte; Fig 5B). At 20 µmol/L of peptide, *NESTIN* and *SOX2* gene expression was increased, while it was not increased at 60 µmol/L. The peptide at both concentrations decreased the expression of the *CD133* marker. The neuron-specific markers *BIII-tubulin* and *DCX* were increased at 60 µmol/L of peptide, indicating a commitment towards the neuronal differentiation pathway. While the astrocyte markers (*GFAP* and *CD44*) were overexpressed (more than 10 times compared to the control condition) with increasing concentrations of peptide, the *GLAST* marker was reduced. The oligodendrocyte marker *CNP* was not modified by the presence of the peptide unlike the *GALC* marker, which was decreased with 60 µmol/L of peptide. Immunofluorescence using antibodies revealed similar effects, but the differences were not as obvious as the RT-qPCR technique because of the very slow growth of these cells (not shown).

## Discussion

The possibility of manipulating NSCs directly in the brain for neurodegenerative therapies would be a major breakthrough in the context of neurological disorders. As previously shown, the NFL-TBS.40-63 peptide is a promising tool to target newborn or adult rat NSCs by modifing their behavior [12]. It is however essential to first evaluate the potential of this peptide to modify human NSCs before considering this strategy as a therapeutic approach. Here, we show that the peptide NFL-TBS.40-63 is able to enter hNSCs by direct translocation, without a major effect on cell viability at low concentrations (below 40 µmol/L). However, above 20 µmol/L, the peptide decreases hNSC proliferation and their capability to form neurospheres, which is consistent with an increase in cell adhesion and differentiation in the neuronal and astrocytic pathways. Taken together, these results show for the first time that a peptide is well internalized in NSCs from human fetuses and it modifies hNSC properties including proliferation and differentiation.

The peptide NFL-TBS.40-63 is able to enter in NSCs from human fetus in a dose-dependent manner as previously shown for NSCs from newborn and adult rats [12] or glioblastoma cells [22, 23]. It was also internalized by oligodendrocytes [24] and certain human tumor cell lines (HeLa, MCF7, LNCaP) [9, 22], while its internalization was very low in healthy astrocytes or neurons [11, 22]. However, the internalization pathway was not the same for all of these cells. While NFL-TBS.40-63 uptake occurs through clathrin endocytosis in oligodendrocytes [24], or through the three endocytosis pathways in glioblastoma cells [23], it was internalized by direct translocation (an energy and temperature independent pathway), both in human and rat derived NSCs [12]. These different uptake mechanisms could be due to the composition of the membrane. Indeed, interactions between the charges of the peptide and membrane can be affected by membrane components, as already shown for other cell-penetrating peptides [25]. Furthermore, as previously described, the high proliferative activity of glioblastoma cells can account for the massive NFL peptide internalization [23]. Stem cells [26] and tumor cells are also proliferative cells, when compared to neurons [27] or astrocytes [28] that progressively diminish their proliferation rates during differentiation. This observation supports the idea that this peptide at low concentration would be a promising tool to target NSCs of the central nervous system without affecting neurons and astrocytes. Interestingly, the viability of hNSCs was not particularly affected by the peptide, except at high concentration (60 µmol/L), where half of the cells survived. We used Paclitaxel instead of colchicine to study cell viability because colchicine did not significantly affect cell viability, as already reported for other cells like HL-1 cells [29]. The effect of Paclitaxel was not very important, and this could be due to the strong resistance of stem cells to these drugs, as shown previously for mesenchymal stem cells [30].

The NFL-TBS.40-63 peptide has multiple effects on glioblastoma cells where it enters massively. In particular, it destroys their microtubule network, stops the cell cycle, and reduces their proliferation and migration [22]. Here, we show that the microtubule network of hNSCs is not affected by the peptide even at high concentrations, while the microtubule network in glioblastoma cells is affected even at low concentrations (10 µmol/L) [22]. This differential effect on the microtubule network could be explained by a tubulin specifically expressed in glioblastoma cells, which is not present in NSCs. Indeed, docking experiments between the NFL peptide and tubulin indicate that the peptide binds on a site located near the β-tubulin C-terminal end [31]. Moreover, similar docking experiments showed that the conformation of the β-tubulin C-terminal domain is important for the binding of the peptide. Indeed, the C-terminal region of βI tubulin, but not of βIII tubulin, forms internal contacts preventing the binding of the peptide on tubulin [32]. Finally it was shown that β-tubulins are differentially expressed depending on the tissue [33]. The βII-tubulin isotype is found in NSCs [34], while βIII-tubulin is overexpressed in human glioblastoma cells and breast cancer cells [35], where the NFL-TBS.40-63 peptide affects the microtubule network [9]. Interestingly, the NFL-TBS.40-63 peptide does not affect microtubules in mature neurons, which slightly express βIII-tubulin [22]. Taken together, these data suggest that the specific effect of the peptide on microtubules may be due to a specific post-transcriptional modification of the βIII-tubulin, which is present in several cancer cells, but absent in NSCs.

When investigating the effect of the peptide on properties of hNSCs, our data suggest that they lose their stem cell characteristics. Using FACS analysis (Propidium Iodide incorporation) we observed that the cell cycle (G0/G1, G2/M, S phases) was not dramatically modified by the presence of the peptide, but cells were mostly in G2/M and S phases in the presence of peptide (when compared to control conditions). We also measured the incorporation of two thymidine analogues (BrdU and EdU) to investigate the replicative state of cells. Such analogues are incorporated only in the replicative stage of the DNA (during the synthesis stage–phase S). Here, we observed that the peptide increased the incorporation of these analogues after 72 hours. We expected also to have a significant increase in the number of cells in S phase with the FACS technique because of the observed BrdU and EdU increase. This was not the case, and it could be explained by the fact that FACS is a technique, which takes cells in a punctual point compared to BrdU/EdU, where cells are incubated during 5 hours. Cells could have already completed S phase during this period and currently be in G2/M phase or return to G0 phase (differentiated cells for example). Thus, at the time of FACS measurement, BrdU positive cells could be detected as G2/M cells or G0/G1 cells. Moreover, the FACS method cannot distinguish between early and late S phase (late G1 phase and early G2 phase, respectively).

Paradoxically, the presence of the peptide, even at low concentrations (20 µmol/L) in the culture medium of hNSCs during 3 days, induces a significant decrease of the number of hNSC (Trypan blue and Cyquant tests). The effect with 60 µmol/L of peptide was comparable to the effect of colchicine, which indicates a potential toxic effect of the peptide at this high concentration.

When incubated in the right medium (proliferative medium), NSCs form neurospheres in suspension after few days, but fewer neurospheres were formed in the presence of the peptide. These results are consistent with a decrease in the number of cells and an increase in adhesion after 72 hours. Moreover, the size of neurospheres was also reduced as a consequence of the decrease of cell proliferation (number of cells) as soon as 72 hours as previously described [36]. This decrease in size could be explained by the uptake of the NFL peptide in the fast dividing cells as previously shown [23]. Indeed, there are heterogeneous cells in neurospheres, with a small proportion being either slow dividing or fast dividing cells and the proportion of each cell population modifies the size of the neurosphere [37]. We do not know the exact mechanism of inhibition of hNSC proliferation in the long run, but it can be a consequence of the effect of NFL-TBS.40-63 peptide on mitochondria network as previously observed in glioblastoma [38]. Indeed, the mitochondria network is closely linked to the proliferation mechanism thanks to the aspartate synthesis [39], and thus the peptide at low concentration could affect the mitochondria in hNSCs inducing a reduction in proliferation. Moreover, as previously mentioned, the peptide is derived from the tubulin binding site of neuron-specific cytoskeleton protein (neurofilament), which suggests that the peptide has more affinity with neuron-specific tubulin (like βIII tubulin) than other tubulins as previously shown [32]. Since all sequences of β-tubulin are very similar [40], it is possible than the NFL-TBS.40-63 peptide binds other tubulins with less affinity than with the βIII tubulin, resulting in modification of proliferation without affecting the microtubule network.

Several peptides, endogenous or not, were shown to promote proliferation and migration of these cells [41, 42] but none of them had an inhibitory effect on proliferation. To assess whether adhered cells were more differentiated, we studied the expression of several differentiation markers. We first investigated the protein expression of neuronal (CD90) and glial (A2B5 and O4) progenitor markers. We showed an increase expression for neuronal and glial markers in treated cells after 5 days confirming a differentiation towards the neuronal and glial pathways. This technique does not allow discriminating between astrocytic and oligodendrocytic pathways. We further investigated the gene expression of the four pathways (stem cell, neuron, astrocyte and oligodendrocyte). Interestingly, the RT-PCR analysis showed decreased expression of *CD133*, a stem cell specific marker. *NESTIN* and *SOX2* expression were increased after treatment, but both markers are also expressed by neural progenitors [43], suggesting a neuronal commitment of the cells. The mRNA of early neuronal markers Tubulin βIII and *DCX* were both increased at 60 µmol/L of NFL peptide, validating the more advanced neuronal state. *NCAM* which was not modified with the peptide, is expressed particularly by mature neurons, suggesting that cells do not differentiate towards a mature neuronal phenotype [44]. The astrocyte marker *GFAP* was increased with both concentrations of NFL peptide, while *CD44* was only increased at 60 µmol/L, suggesting an engagement towards astrocytic differentiation of the cells. To be sure that we only visualized GFAP expressed by astrocytes, we did not study the delta isoform of *GFAP* which is expressed by neural stem cells [45]. *GLAST* is expressed by fetal stem cells [46], and thus the decrease of *GLAST* expression induced by the peptide could support the loss of stem cell properties. A study has already shown that some human neural stem cells can express the GALC marker [47]. The oligodendrocyte pathway did not seem to be modified by the presence of the peptide in hNSCs. These data explain the expression of *GALC* in our cells and the decreased expression with 60 µmol/L of peptide, which correlated with the loss of stem cell property.

These results could be consistent with a reduction of the number of cells and an increase of adhesion after 72 hours: peptide induces production of differentiated cells, which are post-mitotic cells. We also evaluated whether cells growing in a conditioned medium with peptide could induce differentiation in a specific manner. Even if we had more differentiated cells in the conditioned medium (versus the proliferating medium), the addition of the peptide does not induce cell differentiation in a particular pathway after 10 days in culture. The conditioned medium stimulates the differentiation of cells in the three pathways, and the peptide over-stimulates this differentiation, but without increasing a selective pathway.

In summary, the number of cells was reduced after 3 days of culture as well as the capability to form neurospheres while cell adhesion and differentiation were increased in the presence of peptide. To our knowledge, this is the first report to show that a peptide can penetrate NSCs so efficiently and modify their properties. The peptide has no major effect on these cells at low concentration (less than 20 µm/L), while at higher concentration it inhibits proliferation and the capability to form neurospheres, as well as, increases cell adhesion and differentiation in a proliferative medium. Indeed, all experiments were performed using proliferative medium, and because this peptide is able to stimulate hNSC differentiation in this medium, it would be interesting to counteract the inconvenient transfer of cells in defined medium. Indeed, this peptide could be an interesting tool to target and differentiation endogenous NSCs after intraventricular injection in human patients with neuronal disease, since it is not able to penetrate inside healthy astrocytes or neurons [22]. Further experimentation will be done to describe and detail the mechanism of action for the NFL-TBS.40-63 peptide.

## Conclusions

Previous works showed that the NFL-TBS.40-63 peptide penetrates in NSCs from newborn and adult rats, where it affects their properties, in particular increasing differentiation in neural pathways [12]. Here, we show that this peptide also enters by direct translocation in human NSCs in a dose-dependent manner. The peptide induces a decrease in hNSC proliferation, consistent with an increase in cell adhesion and differentiation in the neuronal and astrocytic pathways. This peptide could be used as a targeting tool following its absorption on nanoparticles as previously shown in glioblastoma cells [48] or rat NSCs [49]. Therefore, this peptide represents a promising new tool to target and manipulate NSCs in the case of neurodegenerative diseases, as well as to bring nanoparticles selectively in such cells.

## Supporting information

S1 Fig **FACS Data for viability (A) and EdU incorporation (B).** Data were created from FCS Express 6 software.
(TIF)

S1 File Raw data supporting the results presented in this investigation. The raw data were collected as described in the “Materials & Methods”and “Results”sections.
(PDF)
