# Supplementary figures and images for: The neurofilament derived-peptide NFL-TBS.40-63 enters in-vitro in human neural stem cells and increases their differentiation
Source: PLoS One. 2018 Aug 9;13(8):e0201578. doi: 10.1371/journal.pone.0201578 (PMC6084907; doi:10.1371/journal.pone.0201578)

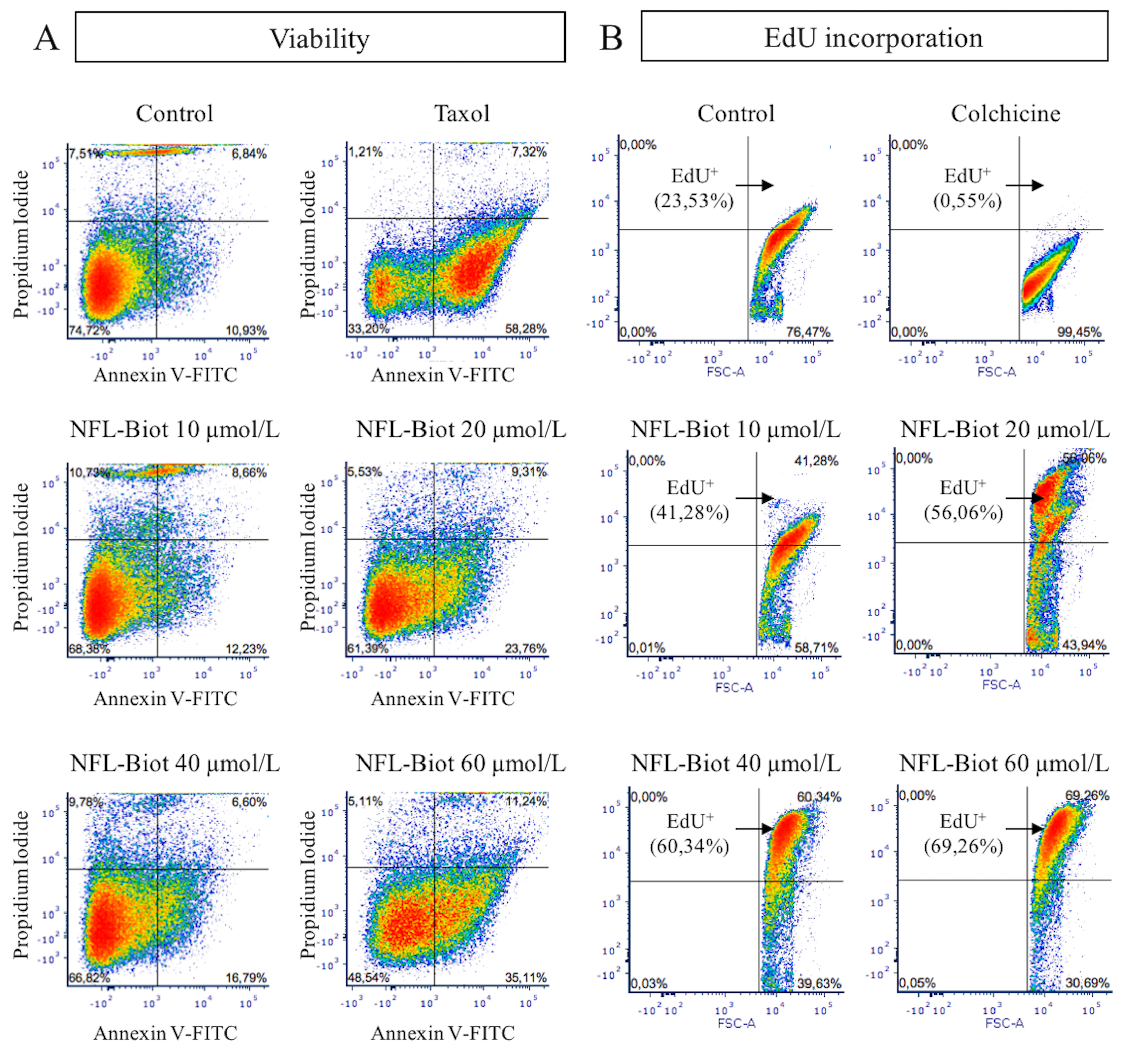

Supplement: S1 Fig — FACS Data for viability (A) and EdU incorporation (B). Data were created from FCS Express 6 software. (TIF) [file pone.0201578.s001.tif]
