# Supplementary material for: The neurofilament derived-peptide NFL-TBS.40-63 enters in-vitro in human neural stem cells and increases their differentiation
Source: PLoS One. 2018 Aug 9;13(8):e0201578. doi: 10.1371/journal.pone.0201578 (PMC6084907; doi:10.1371/journal.pone.0201578)
Supplement: S1 File — The raw data were collected as described in the “Materials & Methods”and “Results”sections. (PDF) [file pone.0201578.s002.pdf]

S1 File: Raw data

Internalisation of the NFL-TBS.40-63 peptide

	Control	4°C	ATP depletion	Chlorpromazin	Nystatin	DMA
	79,54	68,55	85,79	69	72,25	70,19
	74,61	83,13	90,84	81,3	84,98	92,75
	74,83	92,19	89,65	88,3	85,07	80,62
	83,64					
	76,4					
Mean	77,8	81,29	88,76	79,53	80,77	81,19

Tukey's multiple comparisons test	Mean Diff,	95,00% CI of diff,	Significant?	Summary	Adjusted P Value
Control vs. 4°C	-3,486	-17,42 to 10,44	No	ns	0,7618
Control vs. ATP depletion	-10,96	-24,89 to 2,975	No	ns	0,1223
4°C vs. ATP depletion	-7,47	-23,05 to 8,105	No	ns	0,3994

Tukey's multiple comparisons test	Mean Diff,	95,00% CI of diff,	Significant?	Summary	Adjusted P Value
Control vs. Chlorpromazin	-1,729	-19,22 to 15,76	No	ns	0,9898
Control vs. Nystatin	-2,963	-20,45 to 14,53	No	ns	0,9528
Control vs. DMA	-3,383	-20,87 to 14,11	No	ns	0,9323
Chlorpromazin vs. Nystatin	-1,233	-20,79 to 18,32	No	ns	0,9973
Chlorpromazin vs. DMA	-1,653	-21,21 to 17,9	No	ns	0,9935
Nystatin vs. DMA	-0,42	-19,97 to 19,13	No	ns	0,9999

Cell viability

	control	taxol	NFL biot 10 µmol/l	NFL biot 20 µmol/l	NFL biot 40 µmol/l	NFL biot 60 µmol/l
	75,82	45,68	65,36	68,39	68,11	58,73
	68,27	56,48	85,63	67,59	47,61	44,29
	67,89	50,6		74,8	75,26	49,8
	81,03			76,3	84,99	
	95			83,4		
	80,4					
	80,2					
	93,86					
Mean	80,31	50,92	75,5	74,1	68,99	50,94

Tukey's multiple comparisons test	Mean Diff,	95,00% CI of diff,	Significant?	Summary	Adjusted P Value
control vs. colchicine	9,689	-12,01 to 31,39	No	ns	0,8078
control vs. taxol	29,39	7,685 to 51,09	Yes	**	0,0034
control vs. NFL biot 5 µmol/l	14,78	-6,922 to 36,49	No	ns	0,3532
control vs. NFL biot 10 µmol/l	4,814	-20,53 to 30,16	No	ns	0,998
control vs. NFL biot 20 µmol/l	6,213	-12,06 to 24,49	No	ns	0,9431
control vs. NFL biot 40 µmol/l	11,32	-8,315 to 30,95	No	ns	0,5541
control vs. NFL biot 60 µmol/l	29,37	7,665 to 51,07	Yes	**	0,0034
colchicine vs. taxol	19,7	-6,476 to 45,88	No	ns	0,2434
colchicine vs. NFL biot 5 µmol/l	5,093	-21,08 to 31,27	No	ns	0,9976
colchicine vs. NFL biot 10 µmol/l	-4,875	-34,14 to 24,39	No	ns	0,9991
colchicine vs. NFL biot 20 µmol/l	-3,476	-26,89 to 19,94	No	ns	0,9996
colchicine vs. NFL biot 40 µmol/l	1,628	-22,86 to 26,11	No	ns	>0,9999
colchicine vs. NFL biot 60 µmol/l	19,68	-6,496 to 45,86	No	ns	0,2444
taxol vs. NFL biot 5 µmol/l	-14,61	-40,78 to 11,57	No	ns	0,592
taxol vs. NFL biot 10 µmol/l	-24,58	-53,84 to 4,69	No	ns	0,1458
taxol vs. NFL biot 20 µmol/l	-23,18	-46,59 to 0,2362	No	ns	0,0537
taxol vs. NFL biot 40 µmol/l	-18,07	-42,56 to 6,413	No	ns	0,2634
taxol vs. NFL biot 60 µmol/l	-0,02	-26,2 to 26,16	No	ns	>0,9999
NFL biot 5 µmol/l vs. NFL biot 10 µmol/l	-9,968	-39,23 to 19,3	No	ns	0,9425
NFL biot 5 µmol/l vs. NFL biot 20 µmol/l	-8,569	-31,98 to 14,84	No	ns	0,9186
NFL biot 5 µmol/l vs. NFL biot 40 µmol/l	-3,466	-27,95 to 21,02	No	ns	0,9997
NFL biot 5 µmol/l vs. NFL biot 60 µmol/l	14,59	-11,59 to 40,76	No	ns	0,5936
NFL biot 10 µmol/l vs. NFL biot 20 µmol/l	1,399	-25,42 to 28,22	No	ns	>0,9999
NFL biot 10 µmol/l vs. NFL biot 40 µmol/l	6,503	-21,26 to 34,27	No	ns	0,9927
NFL biot 10 µmol/l vs. NFL biot 60 µmol/l	24,56	-4,71 to 53,82	No	ns	0,1464
NFL biot 20 µmol/l vs. NFL biot 40 µmol/l	5,104	-16,4 to 26,61	No	ns	0,9921
NFL biot 20 µmol/l vs. NFL biot 60 µmol/l	23,16	-0,2562 to 46,57	No	ns	0,054
NFL biot 40 µmol/l vs. NFL biot 60 µmol/l	18,05	-6,433 to 42,54	No	ns	0,2646

Microtubles disruption

	control	Colchicine	NFL 20 µmol/l	NFL 60 µmol/l
	13,59	44	10,48	10,53
	1,94	30,67	12,5	12,84
	20	63,11	13,71	27,66
	10,28	15,84	17,92	14,95
	15,84	37,84	19,05	17,82
	12,75		4,85	8,57
	21,31		16,5	
Mean	13,67	38,29	13,57	15,4

Tukey's multiple comparisons test	Mean Diff,	95,00% CI of diff,	Significant?	Summary	Adjusted P Value
control vs. Colchicine	-24,62	-39,9 to -9,335	Yes	**	0,0011
control vs. NFL 20 µmol/l	0,1	-13,85 to 14,05	No	ns	>0,9999

control vs. NFL 60 µmol/l	-1,722	-16,24 to 12,8	No	ns	0,9872
Colchicine vs. NFL 20 µmol/l	24,72	9,435 to 40	Yes	**	0,001
Colchicine vs. NFL 60 µmol/l	22,9	7,092 to 38,7	Yes	**	0,0031
NFL 20 µmol/l vs. NFL 60 µmol/l	-1,822	-16,34 to 12,7	No	ns	0,9849

Cell cycle

G0/G1

	control	Colchicine	NFL biot 10 µmol/l	NFL biot 20 µmol/l	NFL biot 40 µmol/l	NFL biot 60 µmol/l
	73	60,8	67,4	74,8	68,2	71,4
	79,6	56,8	78,2	55,6	77	76,2
	95	61,6	80,8	61,6	67,2	79,2
	86,8	74,6	84,2	78	83,2	79,4
	63	75,6	52,2	63,8	58,8	55
	85,8	79,6		77,2		
Mean	80,53	68,17	72,56	68,5	70,88	72,24

S

	control	Colchicine	NFL biot 10 µmol/l	NFL biot 20 µmol/l	NFL biot 40 µmol/l	NFL biot 60 µmol/l
	22,2	24,8	26,4	20,2	21,2	19,8
	15,4	24,6	14,6	35,6	16,8	20,2
	0	29,4	9,8	16,8	19,4	5,2
	7,2	6,8	5,8	5,4	2,6	9,8
	27,4	9,8	34,4	24,4	37,6	32,6
	9,2	13,4		18		
Mean	13,57	18,13	18,2	20,07	19,52	17,52

G2/M

	control	Colchicine	NFL biot 10 µmol/l	NFL biot 20 µmol/l	NFL biot 40 µmol/l	NFL biot 60 µmol/l
	4,8	14,4	6,2	5	21,2	8,8
	5	18,6	7,2	8,8	6,2	3,6
	5	9	9,4	21,6	13,4	15,6
	6	18,6	10	16,6	14,2	10,8
	9,6	14,6	13,4	11,8	3,6	12,4
	5	3,5		2,4		
Mean	5,9	13,12	9,24	11,03	11,72	10,24

Tukey's multiple comparisons test	Mean Diff,	95,00% CI of diff,	Significant?	Summary	Adjusted P Value
G0/G1					
Control vs. Colchicine	12,37	-3,094 to 27,83	No	ns	0,1924
Control vs. NFL biot 10 µmol/l	7,973	-8,242 to 24,19	No	ns	0,7055
Control vs. NFL biot 20 µmol/l	12,03	-3,428 to 27,49	No	ns	0,2176
Control vs. NFL biot 40 µmol/l	9,653	-6,562 to 25,87	No	ns	0,5114
Control vs. NFL biot 60 µmol/l	8,293	-7,922 to 24,51	No	ns	0,6697
Colchicine vs. NFL biot 10 µmol/l	-4,393	-20,61 to 11,82	No	ns	0,9683
Colchicine vs. NFL biot 20 µmol/l	-0,3333	-15,79 to 15,13	No	ns	>0,9999
Colchicine vs. NFL biot 40 µmol/l	-2,713	-18,93 to 13,5	No	ns	0,9965
Colchicine vs. NFL biot 60 µmol/l	-4,073	-20,29 to 12,14	No	ns	0,9772
NFL biot 10 µmol/l vs. NFL biot 20 µmol/l	4,06	-12,16 to 20,28	No	ns	0,9775
NFL biot 10 µmol/l vs. NFL biot 40 µmol/l	1,68	-15,26 to 18,62	No	ns	0,9997
NFL biot 10 µmol/l vs. NFL biot 60 µmol/l	0,32	-16,62 to 17,26	No	ns	>0,9999
NFL biot 20 µmol/l vs. NFL biot 40 µmol/l	-2,38	-18,6 to 13,84	No	ns	0,9981
NFL biot 20 µmol/l vs. NFL biot 60 µmol/l	-3,74	-19,96 to 12,48	No	ns	0,9844
NFL biot 40 µmol/l vs. NFL biot 60 µmol/l	-1,36	-18,3 to 15,58	No	ns	0,9999
S					
Control vs. Colchicine	-4,567	-20,03 to 10,89	No	ns	0,9543
Control vs. NFL biot 10 µmol/l	-4,633	-20,85 to 11,58	No	ns	0,9603
Control vs. NFL biot 20 µmol/l	-6,5	-21,96 to 8,961	No	ns	0,8223
Control vs. NFL biot 40 µmol/l	-5,953	-22,17 to 10,26	No	ns	0,8912
Control vs. NFL biot 60 µmol/l	-3,953	-20,17 to 12,26	No	ns	0,98
Colchicine vs. NFL biot 10 µmol/l	-0,06667	-16,28 to 16,15	No	ns	>0,9999
Colchicine vs. NFL biot 20 µmol/l	-1,933	-17,39 to 13,53	No	ns	0,9991
Colchicine vs. NFL biot 40 µmol/l	-1,387	-17,6 to 14,83	No	ns	0,9999
Colchicine vs. NFL biot 60 µmol/l	0,6133	-15,6 to 16,83	No	ns	>0,9999
NFL biot 10 µmol/l vs. NFL biot 20 µmol/l	-1,867	-18,08 to 14,35	No	ns	0,9994
NFL biot 10 µmol/l vs. NFL biot 40 µmol/l	-1,32	-18,26 to 15,62	No	ns	>0,9999
NFL biot 10 µmol/l vs. NFL biot 60 µmol/l	0,68	-16,26 to 17,62	No	ns	>0,9999
NFL biot 20 µmol/l vs. NFL biot 40 µmol/l	0,5467	-15,67 to 16,76	No	ns	>0,9999
NFL biot 20 µmol/l vs. NFL biot 60 µmol/l	2,547	-13,67 to 18,76	No	ns	0,9974
NFL biot 40 µmol/l vs. NFL biot 60 µmol/l	2	-14,94 to 18,94	No	ns	0,9993
G2/M					
Control vs. Colchicine	-7,217	-22,68 to 8,244	No	ns	0,749
Control vs. NFL biot 10 µmol/l	-3,34	-19,56 to 12,88	No	ns	0,9907
Control vs. NFL biot 20 µmol/l	-5,133	-20,59 to 10,33	No	ns	0,9263
Control vs. NFL biot 40 µmol/l	-5,82	-22,04 to 10,4	No	ns	0,9001
Control vs. NFL biot 60 µmol/l	-4,34	-20,56 to 11,88	No	ns	0,97
Colchicine vs. NFL biot 10 µmol/l	3,877	-12,34 to 20,09	No	ns	0,9817
Colchicine vs. NFL biot 20 µmol/l	2,083	-13,38 to 17,54	No	ns	0,9987
Colchicine vs. NFL biot 40 µmol/l	1,397	-14,82 to 17,61	No	ns	0,9999
Colchicine vs. NFL biot 60 µmol/l	2,877	-13,34 to 19,09	No	ns	0,9953
NFL biot 10 µmol/l vs. NFL biot 20 µmol/l	-1,793	-18,01 to 14,42	No	ns	0,9995
NFL biot 10 µmol/l vs. NFL biot 40 µmol/l	-2,48	-19,42 to 14,46	No	ns	0,9981
NFL biot 10 µmol/l vs. NFL biot 60 µmol/l	-1	-17,94 to 15,94	No	ns	>0,9999
NFL biot 20 µmol/l vs. NFL biot 40 µmol/l	-0,6867	-16,9 to 15,53	No	ns	>0,9999
NFL biot 20 µmol/l vs. NFL biot 60 µmol/l	0,7933	-15,42 to 17,01	No	ns	>0,9999
NFL biot 40 µmol/l vs. NFL biot 60 µmol/l	1,48	-15,46 to 18,42	No	ns	0,9998

Proliferation - Trypan blue

Control	Colchicine	NFL biot 10 µmol/l	NFL biot 20 µmol/l	NFL biot 40 µmol/l	NFL biot 60 µmol/l
99,9	40	125,714	125,71	102,857	17,143
100	34,286	125,714	85,714	57,143	40
100	38,095	57,143	19,048	14,286	9,524
100	93,36735	102,551	39,79592	42,85714	53,57143
100	3,684211	83,87096	65,43779	85,71429	72,81106
100	33,33	43,15789	20	7,631579	0,7894737
100	44,44	77,23	11,88	1,98	0,79
100	9,52	89,11	13,86	2,18	1,19
100	19,05				

	100					
Mean	100	35,09	88,06	47,68	39,33	24,48

Tukey's multiple comparisons test	Mean Diff,	95,00% CI of diff,	Significant?	Summary	Adjusted P Value
Control vs. Colchicine	64,91	24,26 to 105,6	Yes	***	0,0003
Control vs. NFL biot 10 µmol/l	11,94	-30,04 to 53,91	No	ns	0,9569
Control vs. NFL biot 20 µmol/l	52,32	10,34 to 94,29	Yes	**	0,0071
Control vs. NFL biot 40 µmol/l	60,67	18,69 to 102,6	Yes	**	0,0012
Control vs. NFL biot 60 µmol/l	75,52	33,55 to 117,5	Yes	****	<0,0001
Colchicine vs. NFL biot 10 µmol/l	-52,98	-95,97 to -9,978	Yes	**	0,008
Colchicine vs. NFL biot 20 µmol/l	-12,59	-55,59 to 30,4	No	ns	0,9513
Colchicine vs. NFL biot 40 µmol/l	-4,245	-47,24 to 38,75	No	ns	0,9997
Colchicine vs. NFL biot 60 µmol/l	10,61	-32,39 to 53,61	No	ns	0,9766
NFL biot 10 µmol/l vs. NFL biot 20 µmol/l	40,38	-3,863 to 84,62	No	ns	0,0918
NFL biot 10 µmol/l vs. NFL biot 40 µmol/l	48,73	4,486 to 92,97	Yes	*	0,0232
NFL biot 10 µmol/l vs. NFL biot 60 µmol/l	63,58	19,34 to 107,8	Yes	**	0,0013
NFL biot 20 µmol/l vs. NFL biot 40 µmol/l	8,35	-35,89 to 52,59	No	ns	0,993
NFL biot 20 µmol/l vs. NFL biot 60 µmol/l	23,2	-21,04 to 67,45	No	ns	0,6278
NFL biot 40 µmol/l vs. NFL biot 60 µmol/l	14,85	-29,39 to 59,1	No	ns	0,9158

Proliferation - CyQUANT

	Control	Colchicine	NFL biot 10 µmol/l	NFL biot 20 µmol/l	NFL biot 40 µmol/l	NFL biot 60 µmol/l
	368,14		234,43	111,46	62,43	63,85
	297,32		261,18	226,99	125,35	61,36
	243,58		220,08	86,54	69,09	
	113,0857	78,32066	125,4407	139,9327	78,92567	74,68066
	180,1417		146,0257	129,6877	107,1497	100,6827
	230,0067		283,7047	157,9677	160,7077	128,4907
	68,256	41,865	36,216	51,94	39,389	48,017
		61,807	70,947	96,539	77,825	97,39
			60,772	124,615	152,791	79,599
	20,57967	34,62567	77,11867	14,48867	0,9026667	0,8646666
	55,10266	26,46267	50,21067	14,34367	6,604667	1,466667
	54,42367	28,63067	57,56867	13,52367	7,846667	1,141667
	103,2383	111,8423	109,0853	106,5843	107,8413	81,00833
		17,08733	150,1713	148,3833	32,07133	94,47833
	26,86333		22,94133	16,72733	59,60433	27,69633
	82,318	43,146	58,572	46,745	28,909	5,305
	42,827	42,319	27,996	26,307	16,918	4,66
	45,733	47,777	70,071	37,819	17,667	21,519
	115,47	105,225	165,198	83,204	21,548	12,454
	97,646	97,135	102,775	61,16	26,019	7,358
	75,935	77,201	128,513	146,103	40,357	20,976
	74,347	72,609	101,918	88,804	58,233	43,578
	96,465	78,681	113,978	111,602	62,109	33,798
	68,848	75,217	112,225	88,968	51,2	30,197
		42,7	110,43	91,51	93,11	103
		46,83	109,64	79,74	88,56	102,9
Mean	117,2	59,45	115,7	88,53	61,28	49,86

Tukey's multiple comparisons test	Mean Diff,	95,00% CI of diff,	Significant?	Summary	Adjusted P Value
Control vs. Colchicine	57,71	4,286 to 111,1	Yes	*	0,0261
Control vs. NFL biot 10 µmol/l	1,497	-48,01 to 51	No	ns	>0,9999
Control vs. NFL biot 20 µmol/l	28,63	-20,87 to 78,14	No	ns	0,5528
Control vs. NFL biot 40 µmol/l	55,88	6,377 to 105,4	Yes	*	0,0172
Control vs. NFL biot 60 µmol/l	67,3	17,35 to 117,2	Yes	**	0,0021
Colchicine vs. NFL biot 10 µmol/l	-56,22	-107,1 to -5,288	Yes	*	0,0213
Colchicine vs. NFL biot 20 µmol/l	-29,08	-80,01 to 21,85	No	ns	0,5668
Colchicine vs. NFL biot 40 µmol/l	-1,829	-52,76 to 49,1	No	ns	>0,9999
Colchicine vs. NFL biot 60 µmol/l	9,588	-41,77 to 60,94	No	ns	0,9944
NFL biot 10 µmol/l vs. NFL biot 20 µmol/l	27,14	-19,66 to 73,93	No	ns	0,55
NFL biot 10 µmol/l vs. NFL biot 40 µmol/l	54,39	7,588 to 101,2	Yes	*	0,0127
NFL biot 10 µmol/l vs. NFL biot 60 µmol/l	65,8	18,54 to 113,1	Yes	**	0,0013
NFL biot 20 µmol/l vs. NFL biot 40 µmol/l	27,25	-19,55 to 74,05	No	ns	0,5453
NFL biot 20 µmol/l vs. NFL biot 60 µmol/l	38,67	-8,597 to 85,93	No	ns	0,1762
NFL biot 40 µmol/l vs. NFL biot 60 µmol/l	11,42	-35,85 to 58,68	No	ns	0,9819

BrdU incorporation

	Control	Colchicine	NFL Biot 10 µmol/l	NFL Biot 20 µmol/l	NFL Biot 40 µmol/l	NFL Biot 60 µmol/l
	0,135	0,091	0,343	0,683	0,482	0,591
	0,202	0,063	0,107	0,853	0,399	0,32
	0,102	0,125	0,141	0,489	0,464	0,493
	-0,023	-0,039	0,578	0,704	0,668	0,506
	0,033	-0,065	0,584	0,519	0,684	0,84
	0,049	-0,044	0,392	0,254	0,452	0,296
Mean	1	-1,139	9,365	2,653	1,22	0,92

Tukey's multiple comparisons test	Mean Diff,	95,00% CI of diff,	Significant?	Summary	Adjusted P Value
Control vs. Colchicine	2,139	-7,669 to 11,95	No	ns	0,9853
Control vs. NFL Biot 10 µmol/l	-8,365	-18,17 to 1,444	No	ns	0,1317
Control vs. NFL Biot 20 µmol/l	-1,653	-16,64 to 13,33	No	ns	0,9994
Control vs. NFL Biot 40 µmol/l	-0,22	-17,9 to 17,46	No	ns	>0,9999
Control vs. NFL Biot 60 µmol/l	0,08	-17,6 to 17,76	No	ns	>0,9999
Colchicine vs. NFL Biot 10 µmol/l	-10,5	-20,31 to -0,6953	Yes	*	0,03
Colchicine vs. NFL Biot 20 µmol/l	-3,792	-18,77 to 11,19	No	ns	0,9717
Colchicine vs. NFL Biot 40 µmol/l	-2,359	-20,04 to 15,32	No	ns	0,9985
Colchicine vs. NFL Biot 60 µmol/l	-2,059	-19,74 to 15,62	No	ns	0,9992
NFL Biot 10 µmol/l vs. NFL Biot 20 µmol/l	6,711	-8,271 to 21,69	No	ns	0,7543
NFL Biot 10 µmol/l vs. NFL Biot 40 µmol/l	8,145	-9,538 to 25,83	No	ns	0,7324
NFL Biot 10 µmol/l vs. NFL Biot 60 µmol/l	8,445	-9,238 to 26,13	No	ns	0,7022
NFL Biot 20 µmol/l vs. NFL Biot 40 µmol/l	1,433	-19,56 to 22,43	No	ns	>0,9999
NFL Biot 20 µmol/l vs. NFL Biot 60 µmol/l	1,733	-19,26 to 22,73	No	ns	0,9999
NFL Biot 40 µmol/l vs. NFL Biot 60 µmol/l	0,3	-22,7 to 23,3	No	ns	>0,9999

EdU incorporation

Control	Colchicin	NFL-TBS.40-63 10 M	NFL-TBS.40-63 20 M	NFL-TBS.40-63 40 M	NFL-TBS.40-63 60 M
5,8	0,2	11,8	64,4	13,7	40
48	0,3	23,4	13,5	64	65,8

	42,6	1,4	25	11,7	73,8	84,2
	30,2	0,7	40,6	84,3	10,3	14,4
	17,8		42,4	19,1	86,9	98,2
	3,8		53	91,8	12,3	26,5
			16,3	66	91,1	81,1
					92,2	88,8
Mean	24,7	0,65	30,36	50,11	55,54	62,38

Tukey's multiple comparisons test	Mean Diff,	95,00% CI of diff,	Significant?	Summary	Adjusted P Value
Control vs. Colchicine	24,05	-30,68 to 78,78	No	ns	0,7686
Control vs. NFL biot 10 µmol/l	-5,657	-52,83 to 41,52	No	ns	0,9991
Control vs. NFL biot 20 µmol/l	-25,41	-72,59 to 21,76	No	ns	0,5877
Control vs. NFL biot 40 µmol/l	-30,84	-76,63 to 14,95	No	ns	0,3459
Control vs. NFL biot 60 µmol/l	-37,68	-83,47 to 8,117	No	ns	0,1576
Colchicine vs. NFL biot 10 µmol/l	-29,71	-82,85 to 23,44	No	ns	0,5492
Colchicine vs. NFL biot 20 µmol/l	-49,46	-102,6 to 3,68	No	ns	0,0802
Colchicine vs. NFL biot 40 µmol/l	-54,89	-106,8 to -2,965	Yes	*	0,0332
Colchicine vs. NFL biot 60 µmol/l	-61,73	-113,6 to -9,802	Yes	*	0,0122
NFL biot 10 µmol/l vs. NFL biot 20 µmol/l	-19,76	-65,08 to 25,56	No	ns	0,7744
NFL biot 10 µmol/l vs. NFL biot 40 µmol/l	-25,18	-69,06 to 18,7	No	ns	0,5212
NFL biot 10 µmol/l vs. NFL biot 60 µmol/l	-32,02	-75,9 to 11,86	No	ns	0,2632
NFL biot 20 µmol/l vs. NFL biot 40 µmol/l	-5,423	-49,31 to 38,46	No	ns	0,999
NFL biot 20 µmol/l vs. NFL biot 60 µmol/l	-12,26	-56,14 to 31,62	No	ns	0,957
NFL biot 40 µmol/l vs. NFL biot 60 µmol/l	-6,838	-49,23 to 35,56	No	ns	0,9963

**Self-renewal**					
Number of spheres					
Control	Colchicine	NFL biot 10 µmol/l	NFL biot 20 µmol/l	NFL biot 40 µmol/l	NFL biot 60 µmol/l
111,11	555,56	333,33	222,22	111,11	111,11
444,44	0	333,33	222,22	222,22	111,11
0	0	444,44	222,22	111,11	222,22
777,78	222,22	444,44	222,22	333,33	111,11
2000	0	555,56	444,44	333,33	0
222,22	111,11	333,33	222,22	333,33	0
222,22	0	333,33	444,44	333,33	0
444,44	222,22	444,44	555,56	111,11	0
666,67	111,11	222,22	444,44	111,11	0
111,11	0	555,56	333,33	222,22	0
444,44	0	444,44	333,33	222,22	0
222,22	222,22	666,67	222,22	111,11	0
444,44	222,22	111,11	333,33	222,22	0
555,56	0	666,67	111,11	222,22	0
333,33	0	333,33	0	0	0
111,11	0	444,44	0	0	0
444,44	229,63	194,44	111,11	111,11	111,11
333,33	153,85	384,62	444,44	111,11	0
555,56	307,69	769,23	222,22	111,11	0
444,44	76,92	769,23	222,22	0	0
444,44	461,54	461,54	111,11	0	0
222,22	384,62	384,62	111,11	0	0
222,22	923,08	153,85	111,11	0	0
444,44	76,92	769,23	222,22	0	0
444,44	461,54	1692,31	444,44	0	0
444,44	307,69	461,54	222,22	0	0
111,11	230,77	461,54	0	0	0
333,33	923,08	384,62	0	0	0
222,22	538,46	538,46	0	15,87	14,81
222,22	153,85	692,31	44,44	307,69	76,92
222,22	76,92	615,38	153,85	230,77	230,77
762,96	153,85	538,46	76,92	76,92	76,92
384,62	230,77	1076,92	153,85	76,92	76,92
384,62	307,69	692,31	76,92	307,69	76,92
307,69	230,77	1076,92	307,69	76,92	153,85
461,54	153,85	461,54	153,85	153,85	153,85
384,62	153,85	538,46	76,92	76,92	76,92
384,62	0	461,54	692,31	76,92	76,92
923,08	0	769,23	307,69	76,92	153,85
307,69	0	769,23	153,85	153,85	76,92
307,69	0	923,08	384,62	307,69	153,85
307,69		846,15	153,85	230,77	230,77
230,77	0	76,92	307,69	153,85	0
461,54	76,92	76,92	384,62	230,77	0
538,46	0	153,85	307,69	153,85	0
307,69	0	0	76,92	0	0
615,38	0	0	307,69	0	0
461,54	0	0	153,85	0	0
384,62	0	0	153,85	0	0
538,46	0	0	76,92	0	0
307,69	0	0	461,54	0	0
615,38	0	0	153,85	0	0
384,62	0	0	153,85	0	153,85
384,62	0	0	307,69	0	76,92
384,62	0	0	76,92	76,92	0
461,54	76,92	76,92	0	0	0
	0	0	0	153,85	0
230,77	0	0	0	0	153,85
76,92	0	153,85	0	0	0
76,92	0	76,92	0	0	0
76,92	0	153,85	0	230,77	0
76,92	0	76,92	0	76,92	0
230,77	0	0	0	0	0
0	0	0	0	230,77	0

	0	76,92	0	0	153,85	76,92
	0	0	230,77	0	76,92	0
	0	76,92	76,92	0	0	0
	76,92	0	76,92	0	76,92	76,92
	230,77	0	0	0	0	0
	538,46	0	0	0	0	0
	0	0	230,77	0	0	0
	153,85	0	0	0	0	0
	76,92	0	230,77	0	0	0
	76,92	0	153,85	0	0	0
	153,85	0	76,92	0	0	0
	846,15	153,85	76,92	0	0	0
	76,92	76,92	76,92	0	0	
	692,31	0	76,92	0	0	
	76,92	76,92	76,92	0	76,92	
	76,92	76,92	230,77		0	
	0	0	153,85		76,92	
	0	76,92	76,92		76,92	
	0	76,92	230,77		0	
	0	76,92	153,85		76,92	
	76,92	153,85	76,92		0	
	0	76,92	76,92		0	
	0	76,92	76,92		0	
	0	230,77	76,92		0	
	76,92	0	153,85			
	0	0	76,92			
	0	0	153,85			
	0	0	76,92			
	0	76,92	76,92			
	692,31	76,92	0			
	615,38	0	0			
	76,92	0	0			
	0	0	0			
	153,85	76,92	0			
	153,85	0	0			
	0	76,92	0			
	153,85	153,85	0			
	76,92	0	0			
	76,92	0				
	0	0				
	76,92	76,92				
	76,92	0				
	0	0				
	0	0				
	0	0				
	76,92	0				
	0	0				
	0	0				
	0	0				
	0	0				
	153,85	0				
	76,92	0				
	76,92	0				
	76,92	0				
	0					
	76,92					
	0					
	76,92					
	76,92					
	153,85					
	0					
	153,85					
	307,69					
	0					
	153,85					
	153,85					
	76,92					
	230,77					
	76,92					
	76,92					
	76,92					
	76,92					
Mean	234,1	87,21	269,4	154,6	84,39	37,31

Tukey's multiple comparisons test	Mean Diff,	95,00% CI of diff,	Significant?	Summary	Adjusted P Value
Control vs. Colchicine	146,9	71,57 to 222,2	Yes	****	<0,0001
Control vs. NFL biot 10 µmol/l	-35,3	-113,5 to 42,9	No	ns	0,7903
Control vs. NFL biot 20 µmol/l	79,45	-4,987 to 163,9	No	ns	0,0787
Control vs. NFL biot 40 µmol/l	149,7	68,02 to 231,4	Yes	****	<0,0001
Control vs. NFL biot 60 µmol/l	196,8	111,3 to 282,3	Yes	****	<0,0001
Colchicine vs. NFL biot 10 µmol/l	-182,2	-262,9 to -101,4	Yes	****	<0,0001
Colchicine vs. NFL biot 20 µmol/l	-67,41	-154,2 to 19,39	No	ns	0,2299
Colchicine vs. NFL biot 40 µmol/l	2,826	-81,29 to 86,94	No	ns	>0,9999
Colchicine vs. NFL biot 60 µmol/l	49,91	-37,91 to 137,7	No	ns	0,5824
NFL biot 10 µmol/l vs. NFL biot 20 µmol/l	114,8	25,41 to 204,1	Yes	**	0,0036
NFL biot 10 µmol/l vs. NFL biot 40 µmol/l	185	98,26 to 271,7	Yes	****	<0,0001
NFL biot 10 µmol/l vs. NFL biot 60 µmol/l	232,1	141,7 to 322,4	Yes	****	<0,0001
NFL biot 20 µmol/l vs. NFL biot 40 µmol/l	70,24	-22,15 to 162,6	No	ns	0,2517
NFL biot 20 µmol/l vs. NFL biot 60 µmol/l	117,3	21,54 to 213,1	Yes	**	0,0066
NFL biot 40 µmol/l vs. NFL biot 60 µmol/l	47,08	-46,27 to 140,4	No	ns	0,7012

Size of sphere					
Control	Colchicine	NFL biot 10 µmol/l	NFL biot 20 µmol/l	NFL biot 40 µmol/l	NFL biot 60 µmol/l
38,67	46,5	222,62	145,12	87,03	86,74
159,54	50,72	196,62	90,32	78	143,58
	27,43	114,82	116,84	65,27	48,58
181,73	35,95	118,27	151,25	118	42,6
112,95	43,33	152,88	98,3	75,2	51,96
69,93	20,92	237,83	84,94	66,99	19,111
170,23	27,56	161,01	109,75	82,4	18,312
143,25	93,68	260,46	140,42	79,23	7,704
187,85	71,42	183,88	146,6	114,85	17,226
104,55	67,85	202,32	187,93	135,77	17,093
158,86	41,32	183,46	101,68	133,57	21,206
142,39	61,25	140,51	156,91	114,83	17,785
218,53	85,86	328,09	147,08	122,32	51,723
184,69	98,54	159,36	93,13	90,42	38,042
158,9	93,38	208,3	105,17	109,39	17,633
92,44	87,83	233,88	94,17	65,85	50,968
	79,37	135,07	106,57	33,92	47,749
127,65	99,12	177,08	181,54	31,285	20,567
239,78	95,98	167,89	160,27	27,003	36,506
305,58	72,65	120,6	187,27	21,152	23,892
223,34	87,22	158,59	39,15	22,713	26,707
333,84	86,34	200,24	199,87	19,868	25,409
186,36	76,82	215,52	128,83	16,688	47,702
265,2	36,556	212,41	107,72	16,092	
62,46	24,821	186,38	81,864	23,244	
211,56	12,147	169,22	45,677	32,557	
258,99	12,865	224,62	43,391	13,697	
143,95	18,032	210,9	45,365	23,186	
374,35	13,973	208,22	68,937	27,55	
400,33	30,112	178,35	35,071	55,25	
406,65	18,959	320,27	116,284	32,299	
226,98	24,865	227,23	41,231	61,948	
100,42	25,501	72,351	59,403	70,116	
80,94	26,228	119,849	49,584	75,292	
109,41	25,3	67,313	37,15	18,075	
72,52	27,806	76,446	38,898	23,509	
91,37	24,484	69,135	52,018	57,676	
60,174	20,361	96,635	43,073	41,858	
86,223	19,483	68,363	72,795	59,642	
53,258	16,776	62,018	40,653	63,15	
100,056	22,921	79,877	47,654	40,711	
78,448	23,881	140,183	51,461	28,2	
79,89	22,806	70,246	57,752	32,723	
80,453	28,08	92,008	36,323	38,525	
101,233	58,709	89,307	49,308	40,856	
55	18,587	66,752	65,683		
70,88	28,008	60,068	43,265		
82,449	29,304	64,687	50,556		
97,722	42,385	55,316	32,659		
75,42	22,177	84,898	24,735		
84,621	28,008	48,488	28,445		
72,035	33,672	65,715	65,084		
102,076	36,585	56,33	28,098		
64,499	34,136	109,969	101,27		
92,751	39,313	58,55	30,02		
84,27	45,289	60,805	25,546		
88,798	23,224	46,045	42,41		
69,868	24,355	87	107,423		
96,792	23,974	88,8	38,037		
107,003	31,993	116,679	49,278		
19,856	22,623	402,168	218,515		
90,315	25,87	71,87	51,198		
88,913	32,268	94,433	83,62		
84,514	27,564	98,108	29,806		
84,816		89,822	21,759		
55,441		66,153	53,976		
162,615		127,268	35,322		
132,288		121,249	34,64		
114,323		90,393	122,439		
86,855		16,294	28,305		
105,531		152,175	45,678		
71,635		109,778	47,123		
77,873		71,868	33,914		
117,556		25,254	75,75		
233,211		62,113	63,197		
23,597		53,387	23,042		
64,182		47,582			
63,785		65,231			
54,247		113,703			
114,98		44,762			
101,123		61,113			
56,98		110,721			
45,245		46,49			
100,272		47,99			
99,701		42,09			
87,121		193,797			
112,799		49,125			
53,915		95,273			
31,372		67,868			
37,907		157,535			
126,565					
45,245					
78,992					
131,522					
93,147					
100,148					
79,255					
42,882					

	39,88					
	42,831					
	89,638					
	75,142					
	74,133					
	25,794					
	33,73					
Mean	115,4	41,49	124,3	77,57	57,51	38,21

Tukey's multiple comparisons test	Mean Diff,	95,00% CI of diff,	Significant?	Summary	Adjusted P Value
Control vs. Colchicine	73,87	46,72 to 101	Yes	****	<0,0001
Control vs. NFL biot 10 µmol/l	-8,934	-33,55 to 15,68	No	ns	0,9044
Control vs. NFL biot 20 µmol/l	37,79	11,99 to 63,58	Yes	***	0,0005
Control vs. NFL biot 40 µmol/l	57,85	27,37 to 88,33	Yes	****	<0,0001
Control vs. NFL biot 60 µmol/l	77,15	37,81 to 116,5	Yes	****	<0,0001
Colchicine vs. NFL biot 10 µmol/l	-82,81	-110,7 to -54,91	Yes	****	<0,0001
Colchicine vs. NFL biot 20 µmol/l	-36,09	-65,03 to -7,145	Yes	**	0,0053
Colchicine vs. NFL biot 40 µmol/l	-16,02	-49,21 to 17,16	No	ns	0,7375
Colchicine vs. NFL biot 60 µmol/l	3,277	-38,2 to 44,75	No	ns	>0,9999
NFL biot 10 µmol/l vs. NFL biot 20 µmol/l	46,72	20,14 to 73,3	Yes	****	<0,0001
NFL biot 10 µmol/l vs. NFL biot 40 µmol/l	66,78	35,64 to 97,93	Yes	****	<0,0001
NFL biot 10 µmol/l vs. NFL biot 60 µmol/l	86,08	46,23 to 125,9	Yes	****	<0,0001
NFL biot 20 µmol/l vs. NFL biot 40 µmol/l	20,06	-12,03 to 52,15	No	ns	0,473
NFL biot 20 µmol/l vs. NFL biot 60 µmol/l	39,36	-1,235 to 79,96	No	ns	0,0634
NFL biot 40 µmol/l vs. NFL biot 60 µmol/l	19,3	-24,43 to 63,03	No	ns	0,8044

Cell adhesion

	Control	Colchicine	NFL biot 10 µmol/l	NFL biot 20 µmol/l	NFL biot 40 µmol/l	NFL biot 60 µmol/l
	405,468	336,6187	439,031	484,88	385,48	272,123
	345,659	249,4327	360,191	457,469	359,939	268,698
	465,57	113,7427	472,684	503,203	382,339	257,754
	173,2877	146,498	236,2207	304,5767	408,2337	419,1597
	274,7447	110,879	227,1657	379,2987	354,8917	332,8317
	189,7417	108,697	197,3227	215,542	234,2787	285,5857
	202,681	39,061	144,717	119,181	131,834	178,6
	201,127	44,578	171,82	274,531	125,402	123,451
	101,491	42,539	122,622	136,686	554,726	119,25
	123,455	84,83	198,684	158,505	543,576	448,316
	165,901	83,377	109,831	263,109	469,808	461,576
	214,425	228,834	245,941	319,726	551,4	596,194
	201,583	117,286	223,063	176,95	542,731	645,389
	281,409	157,981	357,987	338,398	593,024	579,079
	279,288		392,859	308,412	341,247	750,008
	362,508		433,717	308,054	365,227	417,546
	429,073				389,751	438,961
Mean	259,8	133,2	270,9	296,8	396,1	393,1

Tukey's multiple comparisons test	Mean Diff,	95,00% CI of diff,	Significant?	Summary	Adjusted P Value
Control vs. Colchicine	126,7	-10,53 to 263,9	No	ns	0,0876
Control vs. NFL biot 10 µmol/l	-11,02	-143,4 to 121,4	No	ns	0,9999
Control vs. NFL biot 20 µmol/l	-36,93	-169,4 to 95,49	No	ns	0,9647
Control vs. NFL biot 40 µmol/l	-136,3	-266,7 to -5,861	Yes	*	0,0352
Control vs. NFL biot 60 µmol/l	-133,3	-261,9 to -4,724	Yes	*	0,0376
Colchicine vs. NFL biot 10 µmol/l	-137,7	-276,8 to 1,436	No	ns	0,0541
Colchicine vs. NFL biot 20 µmol/l	-163,6	-302,7 to -24,48	Yes	*	0,0116
Colchicine vs. NFL biot 40 µmol/l	-262,9	-400,2 to -125,7	Yes	****	<0,0001
Colchicine vs. NFL biot 60 µmol/l	-260	-395,5 to -124,5	Yes	****	<0,0001
NFL biot 10 µmol/l vs. NFL biot 20 µmol/l	-25,92	-160,3 to 108,5	No	ns	0,9932
NFL biot 10 µmol/l vs. NFL biot 40 µmol/l	-125,2	-257,7 to 7,179	No	ns	0,0747
NFL biot 10 µmol/l vs. NFL biot 60 µmol/l	-122,3	-252,9 to 8,345	No	ns	0,0801
NFL biot 20 µmol/l vs. NFL biot 40 µmol/l	-99,33	-231,8 to 33,1	No	ns	0,2556
NFL biot 20 µmol/l vs. NFL biot 60 µmol/l	-96,37	-227 to 34,26	No	ns	0,2728
NFL biot 40 µmol/l vs. NFL biot 60 µmol/l	2,961	-125,6 to 131,5	No	ns	>0,9999

Differentiation - FACS

CD90			
	Control	NFL 20 µmol/l	NFL 60 µmol/l
Proliferative medium	0,01	4,1	9,14
	0,02	2,56	27,22
	0,17	4,33	26,72
	0,13		9,48
			20,7
Mean	0,0825	3,663	18,65
Conditionned medium	2,2	6,6	27,58
	0,28	15,58	19,82
	1,4	26,75	
	5	12,25	33,74
Mean	2,22	15,3	27,05

Tukey's multiple comparisons test	Mean Diff,	95,00% CI of diff,	Significant?	Summary	Adjusted P Value
		Proliferative medium			
Control vs. NFL	-3,581	-15,65 to 8,486	No	ns	0,7311
Control vs. NFL	-18,57	-29,17 to -7,971	Yes	***	0,0009
NFL vs. NFL	-14,99	-26,53 to -3,45	Yes	*	0,0104
		Conditionned medium			
Control vs. NFL	-13,08	-24,25 to -1,903	Yes	*	0,0208
Control vs. NFL	-24,83	-36,89 to -12,76	Yes	***	0,0002
NFL vs. NFL	-11,75	-23,82 to 0,3154	No	ns	0,057

Sidak's multiple comparisons test	Mean Diff,	95,00% CI of diff,	Significant?	Summary	Adjusted P Value
		Proliferative medium - Conditionned medium			
Control	-2,138	-13,66 to 9,389	No	ns	0,9493
NFL	-11,63	-24,08 to 0,8186	No	ns	0,071
NFL	-8,395	-20,3 to 3,51	No	ns	0,2196

A2B5			
	Control	NFL 20 µmol/l	NFL 60 µmol/l
Proliferative medium	0,76	2,03	24,31
	0,76	7,39	40,02
	2,06	2,69	26,3
Mean	1,193	4,037	30,21
Conditionned medium	1	30,76	23,93
	7,17	18,54	37,99
	8,61	16,54	23,69
Mean	5,593	21,95	28,54

Tukey's multiple comparisons test	Mean Diff,	95,00% CI of diff,	Significant?	Summary	Adjusted P Value
Proliferative medium					
Control vs. NFL	-2,843	-16,18 to 10,49	No	ns	0,8389
Control vs. NFL	-29,02	-42,35 to -15,68	Yes	***	0,0002
NFL vs. NFL	-26,17	-39,51 to -12,84	Yes	***	0,0006
Conditionned medium					
Control vs. NFL	-16,35	-29,69 to -3,017	Yes	*	0,017
Control vs. NFL	-22,94	-36,28 to -9,607	Yes	**	0,0017
NFL vs. NFL	-6,59	-19,93 to 6,747	No	ns	0,4123

Sidak's multiple comparisons test	Mean Diff,	95,00% CI of diff,	Significant?	Summary	Adjusted P Value
Proliferative medium - Conditionned medium					
Control	-4,4	-18,25 to 9,449	No	ns	0,7797
NFL	-17,91	-31,76 to -4,061	Yes	*	0,0112
NFL	1,673	-12,18 to 15,52	No	ns	0,9831

O4			
	Control	NFL 20 µmol/l	NFL 60 µmol/l
Proliferative medium	0,35	11,5	19,56
	1,99	14,2	30,6
	0,2	5,71	22,04
Mean	0,8467	10,47	24,07
Conditionned medium	0,88	19,59	42,47
	3,38	16,88	65,92
	7,6	21,62	49,51
Mean	3,953	19,36	52,63

Tukey's multiple comparisons test	Mean Diff,	95,00% CI of diff,	Significant?	Summary	Adjusted P Value
Proliferative medium					
Control vs. NFL	-9,623	-22,67 to 3,426	No	ns	0,1628
Control vs. NFL	-23,22	-36,27 to -10,17	Yes	**	0,0013
NFL vs. NFL	-13,6	-26,65 to -0,547	Yes	*	0,041
Conditionned medium					
Control vs. NFL	-15,41	-28,46 to -2,36	Yes	*	0,0212
Control vs. NFL	-48,68	-61,73 to -35,63	Yes	****	<0,0001
NFL vs. NFL	-33,27	-46,32 to -20,22	Yes	****	<0,0001

Sidak's multiple comparisons test	Mean Diff,	95,00% CI of diff,	Significant?	Summary	Adjusted P Value
Proliferative medium - Conditionned medium					
Control	-3,107	-16,66 to 10,44	No	ns	0,9009
NFL	-8,893	-22,44 to 4,657	No	ns	0,2565
NFL	-28,57	-42,12 to -15,02	Yes	***	0,0002

Differentiation - qPCR

	Stem cells markers					
	NFL Biot 20 mol/l			NFL Biot 60 mol/l		
	CD133	NESTIN	SOX2	CD133	NESTIN	SOX2
	0,6113201	1,71119	1,29684	0,2726269	0,9075192	0,528509
	0,2952482	1,283426	0,8438158	0,31	1,47	1,39
	0,69	1,43	1,29	0,3977682	0,99	1,05
	0,61	1,92	1,58		1,079228	1,552938
	0,4537596	1,771535	1,469169			
Mean	0,5321	1,623	1,296	0,3268	1,112	1,13

Sidak's multiple comparisons test	Mean Diff,	95,00% CI of diff,	Significant?	Summary	Adjusted P Value
NFL Biot 20 mol/l - NFL Biot 60 mol/l					
CD133	0,2053	-0,3147 to 0,7252	No	ns	0,6801
Nestin	0,5115	0,03396 to 0,9891	Yes	*	0,0335
Sox2	0,1656	-0,312 to 0,6432	No	ns	0,7584

	Neuronal markers					
	NFL Biot 20 mol/l			NFL Biot 60 mol/l		
	TUBBIII	DCX	NCAM	TUBBIII	DCX	NCAM
	0,7295102	0,7955365	1,101905	2,305373	3,944931	1,898684
	0,8321987	0,9592641	0,8207416	0,8379871	0,6439408	0,2474142
	0,94	1,82	0,94	1,6	3,25	0,97
	1,62	1,41	0,83	3,34	0,72	0,35
	0,9760318	1,618884	0,8555951	2,73208	1,972465	1,209994
Mean	1,02	1,321	0,9096	2,163	2,106	0,9352

Sidak's multiple comparisons test	Mean Diff,	95,00% CI of diff,	Significant?	Summary	Adjusted P Value
NFL Biot 20 mol/l vs. NFL Biot 60 mol/l					
BIII	-1,144	-48,23 to 45,94	No	ns	>0,9999
DCX	-0,7855	-47,87 to 46,3	No	ns	>0,9999
NCAM	-0,02557	-47,11 to 47,06	No	ns	>0,9999

	Astrocyte markers					
	NFL Biot 20 mol/l			NFL Biot 60 mol/l		
	GFAP	CD44	GLAST	GFAP	CD44	GLAST
	3,000078	0,9043794	0,6574714	220,5558	4,958831	0,1587689
	26,72281	0,7144971	0,2773924	63,77858	2,34567	0,5
	28,2465	1,21	0,95		4,01	0,28
		0,9	0,92		5,53	0,2822411
		1,574616	0,823591		6,04189	
Mean	19,32	1,061	0,7257	142,2	4,577	0,3053

Sidak's multiple comparisons test	Mean Diff,	95,00% CI of diff,	Significant?	Summary	Adjusted P Value
NFL Biot 20 mol/l - NFL Biot 60 mol/l					
GFAP	-122,8	-186,6 to -59,05	Yes	***	0,0002
CD44	-3,517	-47,72 to 40,68	No	ns	0,9956

GLAST	0,4204	-46,46 to 47,3	No	ns	>0,9999

	Oligodendrocyte markers			
	NFL Biot 20 mol/l		NFL Biot 60 mol/l	
	GALC	CNP	GALC	CNP
	1,082975	0,5605831	0,27	1,526259
		0,5843886	0,48	0,5140569
	1,16	1,05	0,4475125	0,94
	1,1	1,09		0,83
	0,9233823	1,144724		0,8122524
Mean	1,067	0,8859	0,3992	0,9245

Sidak's multiple comparisons test	Mean Diff,	95,00% CI of diff,	Significant?	Summary	Adjusted P Value
NFL Biot 20 mol/l - NFL Biot 60 mol/l					
GALC	0,6674	0,1484 to 1,186	Yes	*	0,0127
CNP	-0,03857	-0,4683 to 0,3912	No	ns	0,9691
